# Functional diversification accompanies gene family expansion of *MED2* homologs in *Candida albicans*

**DOI:** 10.1371/journal.pgen.1007326

**Published:** 2018-04-09

**Authors:** Matthew J. Dunn, Griffin M. Kinney, Pamela M. Washington, Judith Berman, Matthew Z. Anderson

**Affiliations:** 1 Department of Microbiology, The Ohio State University, Columbus, OH, United States of America; 2 Department of Molecular Microbiology and Biotechnology, Tel Aviv University, Tel Aviv, Israel; 3 Department of Microbial Infection and Immunity, The Ohio State University, Columbus, OH, United States of America; Duke University Medical Center, UNITED STATES

## Abstract

Gene duplication facilitates functional diversification and provides greater phenotypic flexibility to an organism. Expanded gene families arise through repeated gene duplication but the extent of functional divergence that accompanies each paralogous gene is generally unexplored because of the difficulty in isolating the effects of single family members. The telomere-associated (*TLO*) gene family is a remarkable example of gene family expansion, with 14 members in the more pathogenic *Candida albicans* relative to two *TLO* genes in the closely-related species *C*. *dubliniensis*. *TLO* genes encode interchangeable Med2 subunits of the major transcriptional regulatory complex Mediator. To identify biological functions associated with each *C*. *albicans TLO*, expression of individual family members was regulated using a Tet-ON system and the strains were assessed across a range of phenotypes involved in growth and virulence traits. All *TLO*s affected multiple phenotypes and a single phenotype was often affected by multiple *TLO*s, including simple phenotypes such as cell aggregation and complex phenotypes such as virulence in a *Galleria mellonella* model of infection. No phenotype was regulated by all *TLO*s, suggesting neofunctionalization or subfunctionalization of ancestral properties among different family members. Importantly, regulation of three phenotypes could be mapped to individual polymorphic sites among the *TLO* genes, including an indel correlated with two phenotypes, growth in sucrose and macrophage killing. Different selective pressures have operated on the *TLO* sequence, with the 5’ conserved Med2 domain experiencing purifying selection and the gene/clade-specific 3’ end undergoing extensive positive selection that may contribute to the impact of individual *TLO*s on phenotypic variability. Therefore, expansion of the *TLO* gene family has conferred unique regulatory properties to each paralog such that it influences a range of phenotypes. We posit that the genetic diversity associated with this expansion contributed to *C*. *albicans* success as a commensal and opportunistic pathogen.

## Author summary

Gene duplication is a rapid mechanism to generate additional sequences for natural selection to act upon and confer greater organismal fitness. If additional copies of the gene are beneficial, this process may be repeated to produce an expanded gene family containing many copies of related sequences. Following duplication, individual gene family members may retain functions of the ancestral gene or acquire new functions through mutation. How functional diversification accompanies expansion into large gene families remains largely unexplored due to the difficulty in assessing individual genes in the presence of the remaining family members. Here, we addressed this question using an inducible promoter to regulate expression of individual genes of the *TLO* gene family in the commensal yeast and opportunistic pathogen *Candida albicans*, which encode components of a major transcriptional regulator. Induced expression of individual *TLO*s affected a wide range of phenotypes such that significant functional overlap occurred among *TLO* genes and most phenotypes were affected by more than one *TLO*. Induced expression of individual *TLO*s did not produce massive phenotypic effects in most cases, suggesting that functional overlap among *TLO* genes may buffer new mutations that arise. Specific sequence variants among the *TLO* genes correlated with certain phenotypes and these sequence variants did not necessarily correlate with sequence similarity across the entire gene. Therefore, individual *TLO* family members evolved specific functional roles following duplication that likely reflect a combination of inherited function and new mutation.

## Introduction

Changes in gene copy number provide a rapid mechanism of adaptation to new or different environments by utilizing available functional sequences to cope with altered conditions. Gene duplication commonly arises through errors in DNA replication or sister chromatid recombination to produce a second identical gene copy [1–3]. The presence of functionally redundant genes loosens evolutionary constraints on the two paralogs and allows them to mutate through genetic drift [4]. As this process is repeated, gene duplication can lead to gene family expansion, which provides significant evolutionary fodder on which selection can act to promote adaptation.

Following gene duplication, the replicated sequence can either be lost or retained to serve a redundant or new function in the organism. In most cases, one of the paralogs is inactivated by deleterious mutations, thereby restricting further evolution of the other gene duplicate [5, 6]. However, if a mutation in a duplicated gene provides a selective advantage, both paralogs may be retained as they contribute separately to fitness of the organism [7–9]. Accumulated polymorphisms between gene duplicates can lead to subfunctionalization in which each gene performs a separate function that previously existed within the ancestral gene or neofunctionalization where one of the paralogs evolves a novel function and the other retains the ancestral function. Most studies of gene duplication and divergence rely on comparison of two paralogs to assess the selective pressures that operated following gene duplication because it provides a more simplified context for analysis [5, 10–13]. Such copy number variants may have arisen through small scale or whole genome duplication [14–17]. Although the evolutionary outcomes of gene duplication resulting from whole genome duplication have been studied extensively [18–22], small scale duplications are much more common, with copy number variation in some genes occurring at rates up to 1.7x10^-4^ duplications per cell division, far exceeding the basal point mutation rate [23]. The evolutionary fate of genes following small-scale duplication is driven largely by genomic context [24–26], gene dosage and protein complex formation [27–29], as well as by gene expression level [28, 30]. Yet, the evolutionary trajectories of gene families that encode many paralogous sequences remain largely unexplored.

Subtelomeres, or telomere-associated sequences, are genomic regions of linear chromosomes that separate the telomeric repeats from chromosome-specific sequences. These regions typically harbor a mixture of duplicated genes and repetitive sequences that often resemble fragments of mobile genetic elements [31, 32]. Subtelomeric regions evolve rapidly and are characterized by extensive genetic turnover due, in part, to the presence of these repetitive sequences [33, 34]. Frequent recombination, elevated mutation rates via acquisition of single nucleotide polymorphisms (SNPs) and insertions/deletions (indels), and the constant processes of gene duplication and gene disruption contribute to the rapid evolution of subtelomeric regions [25, 35–37]. Consequently, subtelomeres are often the most dynamic regions of the genome [25, 38, 39], with profound changes detectable over time scales readily achieved via experimental evolution studies [36].

Expanded gene families commonly reside within subtelomeric regions and are characterized by extensive copy number variation and a rapid accumulation of mutations that can alter their expression, structure, or function [40]. As a result, gene families that reside within the subtelomeres are typically under strong selection and are associated with species-specific lifestyles that promote organismal success [40–43]. For example, the *MAL*, *MEL*, and *SUC* genes in *S*. *cerevisiae* allow cells to utilize different carbon sources (maltose, melibiose, and sucrose, respectively), and fluctuate in copy number depending on the available growth substrate [40, 44, 45]. In this way, the subtelomeric genes contribute to phenotypic plasticity and rapid adaptation to nutrient availability across diverse environments.

The *Candida* clade of species includes mammalian commensals that are closely related to other *Saccharomycotina* but did not undergo a whole genome duplication event [46, 47]. Of these, *C*. *albicans* is the most clinically prevalent species for humans because it is a common commensal also capable of causing debilitating mucosal infections as well as life-threatening systemic infections [48, 49]. The success of *C*. *albicans* is due, in part, to its ability to occupy and persist in a range of commensal host niches including the gastrointestinal tract (pH 7.4–8, 37–40°C), the oral cavity (pH 6.3–7.4, 33–35°C), and the anaerobic colon [50, 51]. The organism often breaches these mucosal niches and becomes bloodstream-borne, especially in hosts with compromised immunity. Progression of disease is dependent upon host immunity as well as a battery of fungal virulence attributes including the ability to transition between different cell morphologies, to resist stresses within the host including oxidative and cell wall damage, and to evade immune system components [52–57].

The expansion of several gene families involved in virulence traits distinguishes *C*. *albicans* from other *Candida* clade species, and thus may have a role in elevated *C*. *albicans* virulence. Expansion of the *ALS*, *SAP*, and *LIP* gene families in *C*. *albicans* increases the functional capacity of adhesins, proteases, and lipases, respectively, which have known roles in pathogenesis [58–60]. The most dramatic gene expansion occurred within the telomere-associated (*TLO*) gene family, which has fourteen copies in *C*. *albicans*, two copies in the most closely-related *C*. *dubliniensis* species, and a single copy within all other *Candida* species [61, 62]. In *C*. *albicans*, these genes are typically the penultimate gene on each chromosome arm [63, 64]. The fourteen *TLO* genes were classified into three clades (α, β, and γ) based on sequence variation that clusters towards the 3’ end of the gene. *TLO* genes display ~97% nucleotide identity within a clade and 82% identity between clades (when excluding indels), yet the three Tlo clades differ in localization to different cellular compartments and in transcript abundance [63, 64].

All *TLO*s encode a conserved Med2 domain found in the Med2 component of the tail subunit of the Mediator complex. Accordingly, Tloα and Tloβ clade members are functional components of the *C*. *albicans* Mediator complex [65, 66]. Mediator functions as a major transcriptional regulator that recruits RNA polymerase II to specific promoters through interaction with transcription factors [67, 68]. It is unclear if *TLO* expansion has led to functional diversification in *C*. *albicans* and how continued evolution to produce diverse sequences affects functional specialization of the *TLO* genes. More broadly, it is not known how gene family expansion beyond a few members shapes the functional specificity of individual members within the amplified gene family.

Here we investigated the role of individual *TLO* genes across a breadth of biological functions relevant to virulence and to growth under different nutrient conditions. Induced expression of individual *TLO* genes using a Tet-ON approach altered a range of phenotypes including complex interactions such as virulence. In most cases, a phenotype was affected by more than one *TLO* gene, but this effect was not simply a function of *TLO* clade or phylogenetic relatedness. Two phenotypes were associated with specific changes to the Tlo protein sequence at the C-terminal end of the Med2 domain. Furthermore, different evolutionary pressures appear to be operating on the *TLO* gene family, with most polymorphisms encoding synonymous changes in the Med2 region and a vast excess of non-synonymous changes occurring within the gene/clade-specific 3’ end. Thus, expansion of the *TLO* gene family is associated with functional diversification, with significant evidence of selection operating on regions and specific sites within the genes.

## Results

### Construction of Tet-inducible *TLO* strains

Previous experiments have assessed aggregate information on *TLO* function as part of *C*. *albicans* Mediator [66] or for a select few *TLO*s under relatively isolated conditions [62]. Yet, retention of the recently expanded *TLO* gene family across multiple sequenced isolates of *C*. *albicans*, despite the high frequency with which it diverges [36], suggests that individual family members likely provide a selective advantage [69]. To test this hypothesis, we constructed strains in which the expression of individual *TLO* genes could be manipulated through a regulatable promoter, via the Tet-ON inducible expression system designed for use in *C*. *albicans* [70].

**Fig 1:**
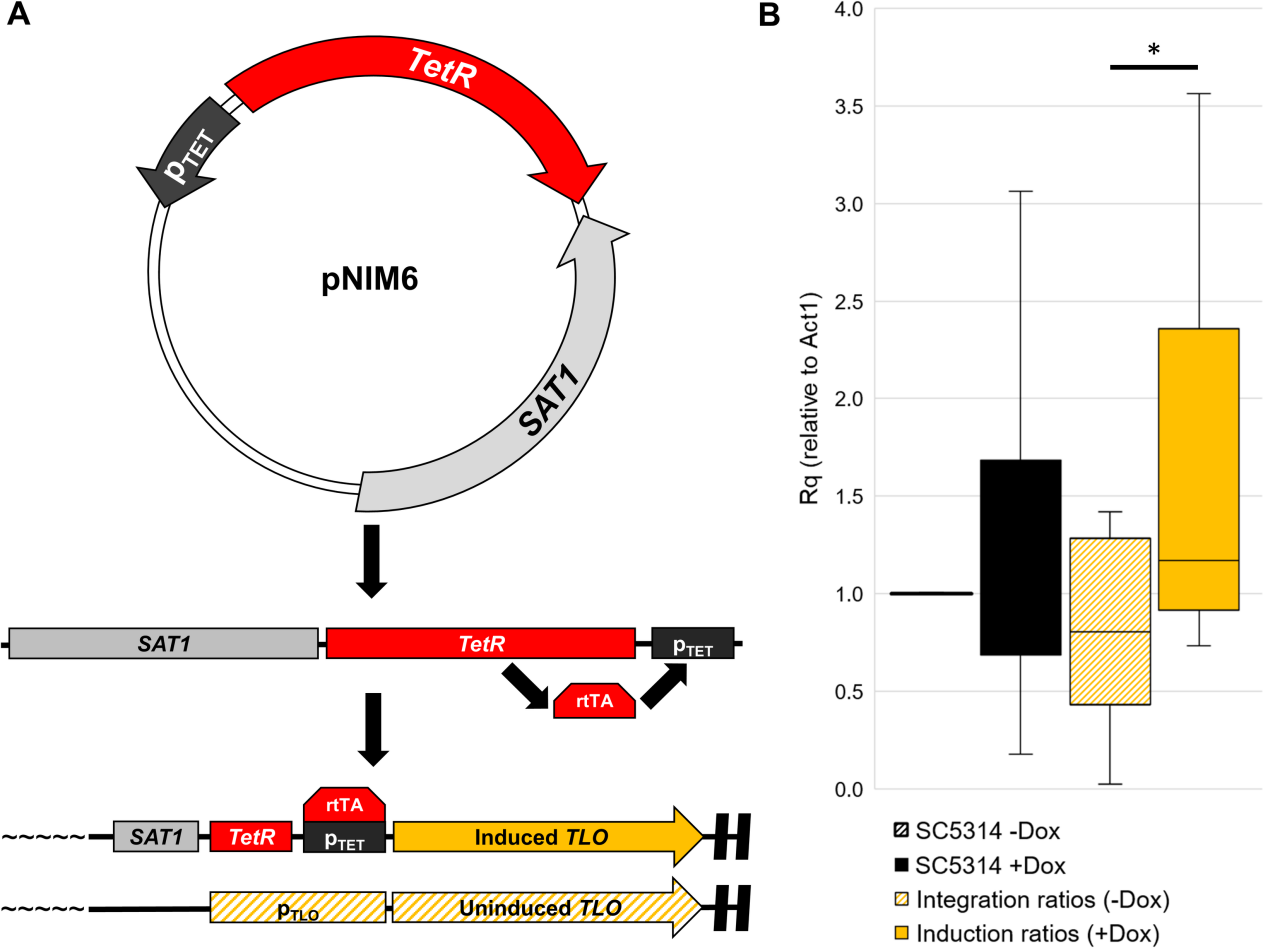
Induced expression of individual *TLO*s by integration of a Tet-ON system. **A.** A segment of pNIM6 was PCR amplified containing a nourseothricin resistance cassette (*SAT1*), a Tet-responsive promoter, and TetR to induce expression. This amplicon was integrated at the endogenous promoter of a variety of *TLO* genes and induction was controlled by the addition of doxycycline (Dox). **B.** Tet-regulated *TLO* strains were grown for 4 hours in the presence or absence of 50 μg/ml Dox and transcript abundance of each regulated *TLO* was determined by qRT-PCR using *ACT1* as a reference gene. Following integration, expression of the Tet-regulated *TLO* was reduced and increased upon induction with Dox. Uninduced and induced expression are shown for either the background strain (black) or a composite of all Tet-regulated strains (gold). * denote p < 0.05.

The Tet responsive promoter (*p*_*TET*_) was targeted to the endogenous locus of individual *TLO* genes where it replaced one of the native promoter alleles (Fig 1A). Integration of the targeting construct produced an in-line inducible expression system in which transcription is activated upon addition of doxycycline (+Dox) and repressed when no Dox (-Dox) is present. In the absence of Dox, only the *TLO* allele lacking the *p*_*TET*_ promoter is expressed. Repeated transformations were performed to produce a series of strains with each strain containing a single Tet-inducible *TLO* gene (S1 Table). Ultimately, we isolated inducible strains for all *TLO*s with the exception of *TLO*α*1* and *TLO*α*10*.

The strains harboring Tet-regulated *TLO*s were then tested for expression in the presence and absence of doxycycline. Primers unique to each *TLO* gene [64] were used to determine the total transcript abundance for individual *TLO*s. Addition of doxycycline to the parental SC5314 strain did not produce any consistent alteration on collective *TLO* gene expression (p = 0.371) (Fig 1B). Integration of the Tet-regulated promoter at *TLO* genes reduced the native expression levels of most targeted loci (Fig 1B, S2 Table), consistent with loss of expression of the regulated allele in the absence of Dox. Induction of the *p*_*TET*_*-TLO* allele by addition of Dox increased transcript abundance significantly for regulated *TLO* genes (p = 0.034) (Figs 1B and S1). Thus, integration of a Tet-regulatable promoter at individual *TLO* loci allows each *TLO* gene to be manipulated and assessed for phenotypic contributions individually.

### Induced *TLO* expression alters *C*. *albicans* growth in different conditions

**Fig 2:**
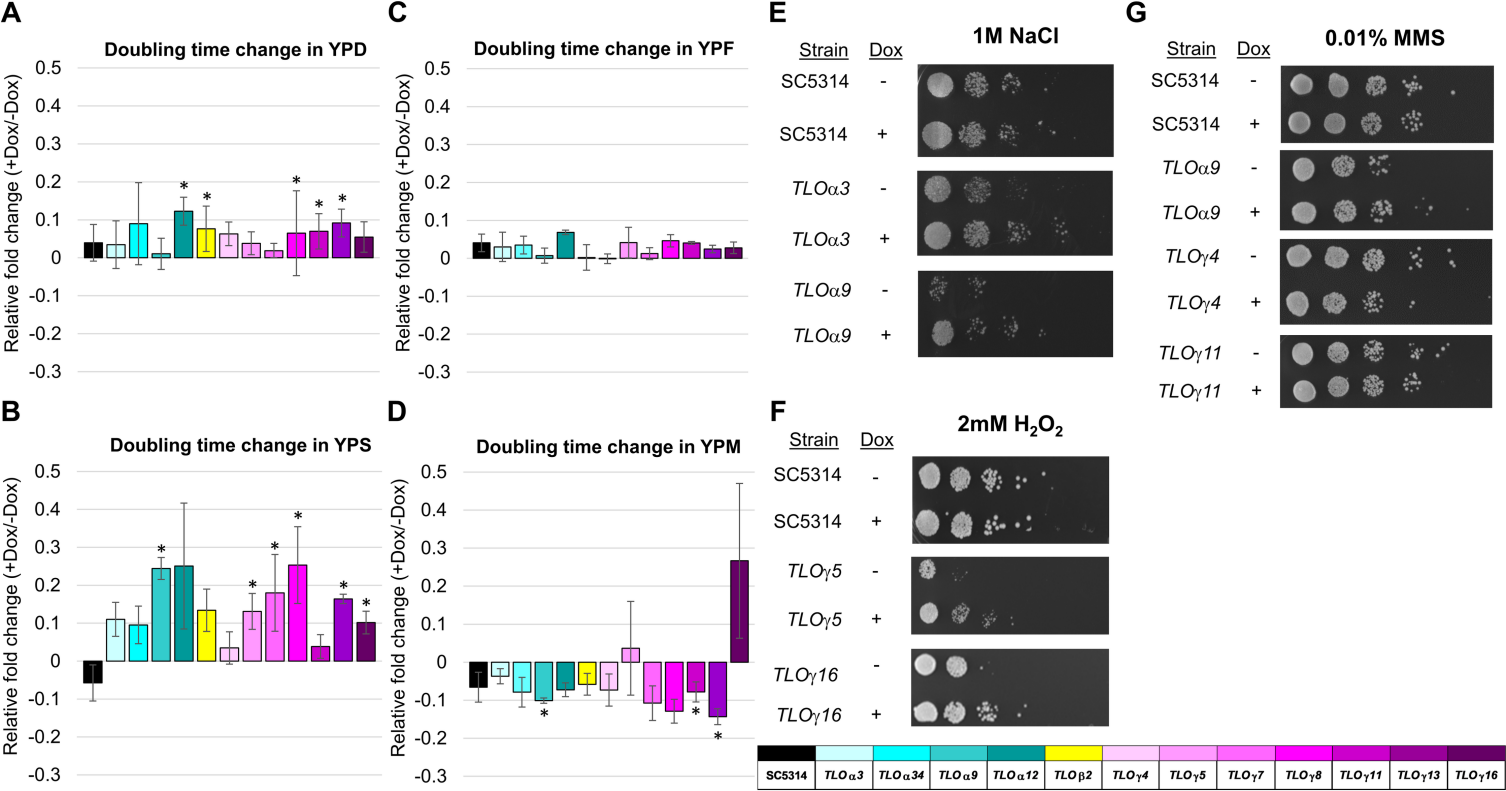
*TLO*s altered growth rates and stress response across a range of conditions. Tet-regulated *TLO* strains were grown overnight in the presence or absence of 50 μg/ml Dox. Cells were diluted 1:2000 and grown in logarithmic phase for 16 hours at 30°C under sustained +/- Dox conditions. Growth media was supplemented with a panel of different carbon sources including dextrose (**A**), sucrose (**B**), fructose (**C**), and maltose (**D**) and the doubling time calculated using a polynomial fit to the growth curve. * denote p < 0.05. For analysis of growth under stress, cells were grown overnight in the presence or absence of 50 μg/ml Dox. Cells were plated at an OD_600_ of 1.0 with ten-fold spot dilutions on SCD solid agar media in the absence of Dox on 1M NaCl (**E**), 2mM H_2_O_2_ (**F**), and 0.01% MMS (**G**). A legend indicates the representative *TLO* gene for each color.

Tlo proteins are incorporated into Mediator, which modulates the expression of a large proportion of the encoded genome [67, 68, 71]. Mediator has previously defined regulatory roles in carbon utilization during growth [72, 73], with *MED2* playing a specific role in gluconeogenesis [74]. To identify alterations in growth that may result from changes in *TLO* expression, doubling times were calculated for all *TLO* inducible strains across a range of nutritional environments. When grown in in rich media conditions (peptides and carbohydrates) with dextrose as the primary carbon source, induction of five different *TLO* genes (*TLO*α*12*, *TLO*β*2*, *TLO*γ*8*, *TLO*γ*11*, and *TLO*γ*13*) increased the observed doubling times, indicating a reduced growth rate relative to uninduced expression of the same strains (Fig 2A). Cells grown with sucrose as the primary carbon source displayed a wider range of doubling times for Tet-induced *TLO* genes, with all strains showing a similar trend towards slower growth (Fig 2B). Six *TLO*s increased doubling times when induced during growth in sucrose with two of these genes also having increased doubling times in dextrose. Little effect on growth rates was observed when cells were cultured in fructose-containing media (Fig 2C). Inclusion of maltose as the primary carbon source had the opposite effect (Fig 2D): most strains grew more rapidly (lower doubling times). Three strains (*TLO*α*9*, *TLO*γ*11*, and *TLO*γ*13*) had significantly faster growth on maltose under inducing conditions. Importantly, addition of Dox to the parental strain had no significant effect on doubling time across the assayed growth condition. These data suggest that there is a complex interplay between growth rates, carbon sources, and the expression of different constellations of *TLO* genes.

Although Tet-induced *TLO* expression affected growth rates across a range of carbon sources in rich media, regulated expression had little effect on growth rates in nutrient-poor media (Spider, YP, or sorbitol) with the notable exception of growth on YP media (0.3% yeast extract, 0.5% peptone), in which growth rates increased for strains with induced expression for six of eight *TLO*s (S2 Fig). The six Tet-regulated *TLO*s that influenced growth in YP included genes that had no effect in YP media supplemented with different sugars, suggesting that the nutrients other than carbon source, such as those in yeast extract, had a different and perhaps stronger effect than did the different carbon sources in rich medium.

To determine if altered expression of the *TLO* genes played a role in response to other stress conditions, we tested growth in the presence of a variety of physiological stresses using spot dilution assays in which the doxycycline used to regulate *TLO* expression had no effect on growth. Similarly, induced *TLO* expression had little effect on growth under several physiological stresses, including growth on synthetic complete defined (SCD) medium at 30°C, 37°C, pH 4.0, pH 8.0, or in the presence of 100μg/mL Calcofluor White (S3 Fig). However, induced expression of *TLO*α*3* and *TLO*α*9* provided a growth advantage relative to –Dox in the presence of 1M NaCl, suggesting that these two alpha-clade *TLO*s confer some resistance to high salt conditions (Fig 2E). By contrast, under oxidative stress conditions, *TLO*s from the gamma-clade provided an advantage in 2mM H_2_O_2_ (Fig 2F). All strains failed to grow well at higher oxidative concentration (6mM H_2_O_2_) and induction of any single *TLO* did not rescue growth (S3C Fig). Induction of *TLO*α*3* expression revealed a growth advantage in the presence of hydroxyurea (HU), a DNA damaging agent (S4 Fig). Conversely, *TLO* induction had more prominent effects in response to methylmalonyl sulfonate (MMS), a different DNA damaging agent. Induction of *TLO* genes both increased resistance to MMS, as was seen with *TLO*α*9*, and reduced resistance with induction of *TLO*γ*4* and *TLO*γ*11* (Fig 2G). Thus, *TLO* genes may influence survival under a range of stress conditions but they appear to play a more prominent role in carbon utilization.

### *TLO*s regulate biofilm-associated phenotypes

**Fig 3:**
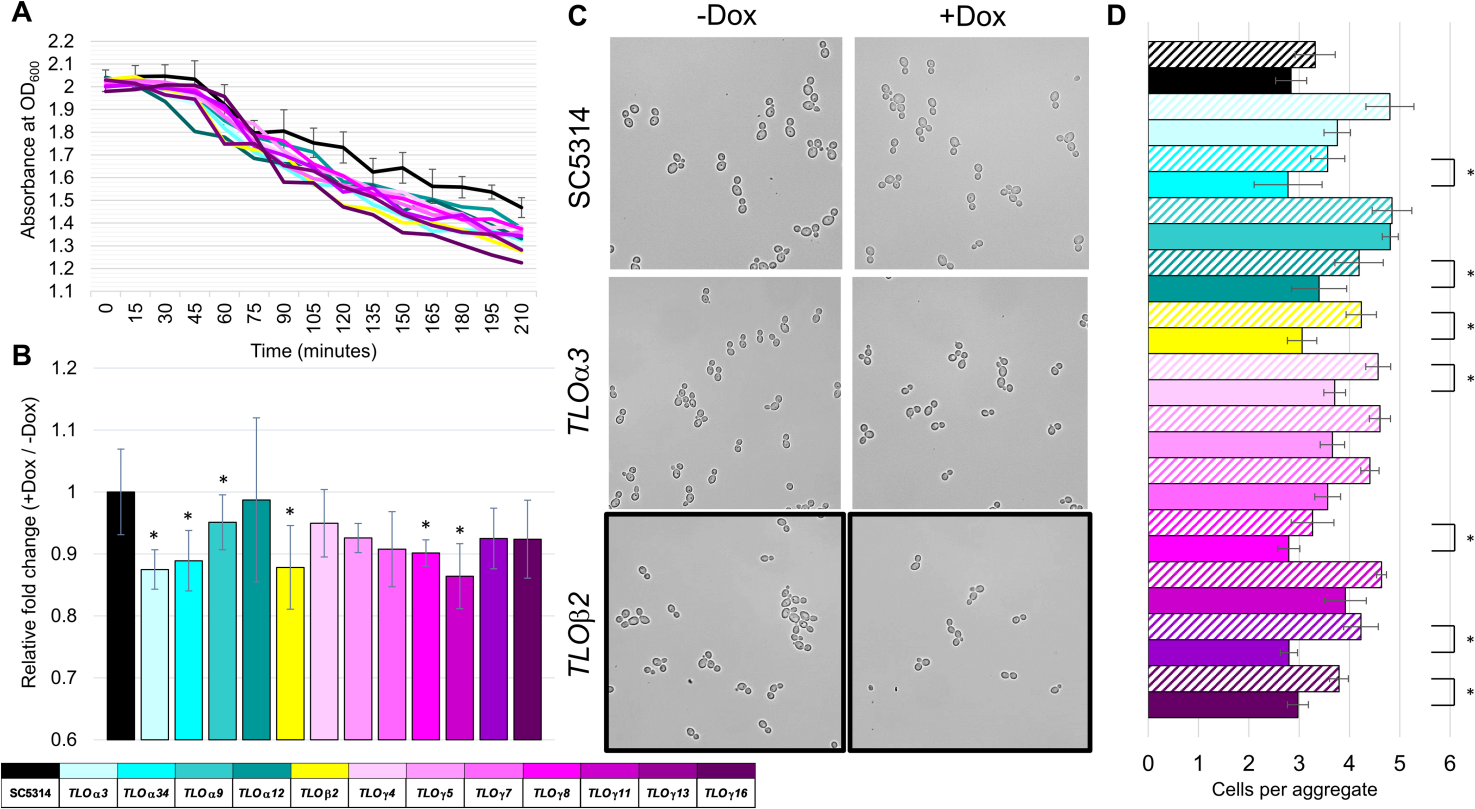
Induction of certain *TLO*s altered flocculation and cell aggregation. **A.** Tet-regulated *TLO* strains were grown overnight in the presence of 50 μg/ml Dox. Vortexed cultures were set to an OD_600_ of 2.0 in a channel cuvette and allowed to settle. Absorbance readings were taken every 15 minutes to measure the rate of flocculation (cell settling). Each data line represents six biological replicates. **B.** Relative fold change of flocculation for +Dox/-Dox is plotted for the 150-minute timepoint where change is normalized relative to SC5314. Error bars indicate standard deviations. **C.** Strains grown overnight in the presence or absence of 50 μg/ml Dox were diluted 1:2 and visualized by light microscopy. **D.** The number of cells per aggregate was quantified for all cells across 10 random fields of view per strain and plotted with standard error bars. A legend indicates the representative *TLO* gene for each color where solid bars indicate +Dox and hatched bars indicate -Dox. * denotes p < 0.05.

Preliminary observations of prepared overnight cultures indicated that cells expressing inducible *TLO*s (supplemented with Dox) were more flocculant because they settled more rapidly when left undisturbed, compared to SC5314 +Dox. A more quantitative analysis of flocculation, in which optical density (OD_600_) of vortexed cells was monitored at 15 min intervals, found that induction of all Tet-regulated *TLO* strains flocculated faster than SC5314 +Dox (Fig 3A). Furthermore, induction of *TLO* expression resulted in faster flocculation relative to the–Dox condition for half of the assayed *TLO* genes (Fig 3B).

Increased flocculation may result from changes in cell size and/or cell aggregation. Neither introduction of the *p*_*TET*_ promoter nor induction of *TLO* expression for any strain caused a noticeable change in cell size (S5 Fig). Conversely, induced *TLO* expression significantly altered cell aggregation. Whereas SC5314 formed aggregates composed of roughly equal numbers of cells in the presence or absence of Dox, induction of *TLO* expression significantly decreased aggregate size for seven of twelve *TLO*s (Fig 3C and 3D). Reduced aggregate size would be expected to decrease the degree of flocculation, because larger aggregates should settle more rapidly. This suggests that additional factors likely contribute to the enhanced cell settling phenotype in Tet-induced *TLO* strains.

**Fig 4:**
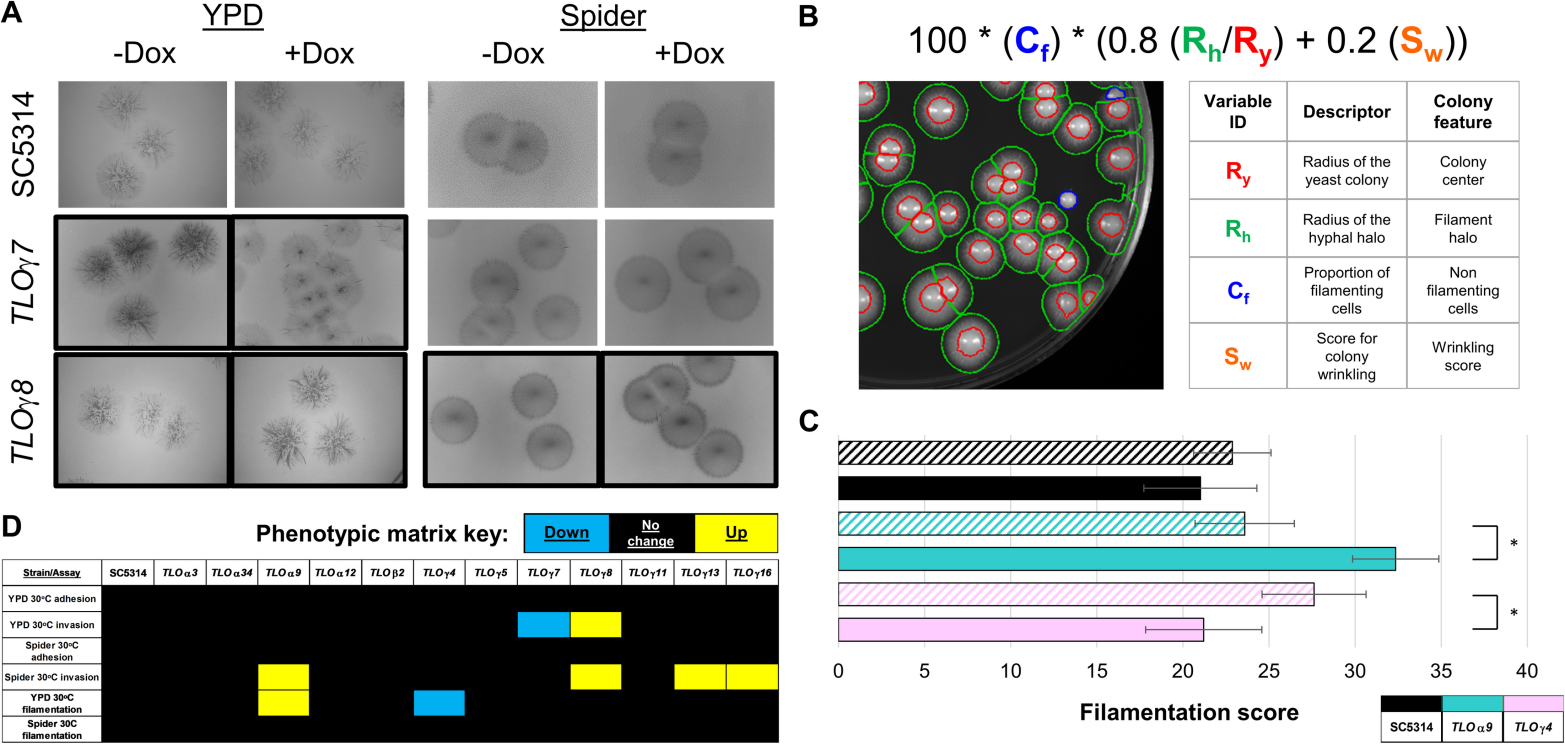
*TLO*s regulate filamentation and agar invasion. *TLO* induction strains were grown for five days following incubation overnight in the presence or absence of 50 μg/ml Dox. **A.** After five days of growth at 30°C on either YPD or Spider solid media, colonies were washed off and the extent of filamentous invasion was profiled. Black image borders indicate phenotypic changes between +/–Dox conditions. **B.** Following seven days of growth at 30°C on either YPD or Spider solid media, surface filamentation was imaged for the whole plate. Analysis was performed on all colonies using the given equation to score filamentation for each colony. **C.** The two *TLO*s with significant changes in filamentation between +Dox and–Dox are shown along with the SC5314 control. **D.** Significant changes due to growth in the presence of Dox versus–Dox are visualized as a heatmap for adhesion, invasion, and colony filamentation on both YPD and Spider solid media at 30°C. Blue indicates a decrease in phenotype, black indicated no phenotype change, and yellow indicates an increase in phenotype. All experiments were performed with a minimum of three replicates. A legend indicates the representative *TLO* gene for each color where solid bars indicate +Dox and hatched bars indicate -Dox. * denotes p < 0.05.

Filamentous growth can contribute to flocculation by both increasing cell size and altering the surface properties of *C*. *albicans* cells, such that they adhere to one another more readily [75, 76]. To test the degree to which filamentous growth affected flocculation, we performed solid agar adhesion-invasion assays for all Tet-regulated *TLO* strains. No tight adhesion to solid YPD or Spider media at 30°C was detected for any of the strains under any condition (S6 Fig). However, Tet-induced *TLO* expression did influence the degree of agar invasion as measured by observable hyphal density and/or prevalence. Induction of *TLO*γ*7* and *TLO*γ*8* decreased and increased, respectively, the extent of agar invasion on YPD at 30°C (Fig 4A and 4D). On solid Spider media at 30°C, increased agar invasion occurred for strains containing four regulated *TLO* genes (*TLO*α*9*, *TLO*γ*8*, *TLO*γ*13*, and *TLO*γ*16* (Fig 4A and 4D).

An alternative approach to assess filamentous growth is to measure a modified M score [69], which quantifies the relative abundance of filamentous growth within a colony’s mass. After 7 days of growth on either YPD or Spider media in the presence of absence of Dox, colonies were imaged and the degree of filamentous growth was measured. Custom scripts assisted in these measurements that differentiate radial filamentous regions of the colony (green) from the central colony body (red) (Fig 4B). This script also accounts for colonies that fail to produce any significant filamentation (blue) in the overall filamentous growth score. As with agar invasion, addition of Dox to SC5314 parental cells did not induce a change in filamentous growth. In contrast, *TLO*α*9* +Dox increased and *TLO*γ*4* +Dox decreased filamentous growth (Figs 4C, 4D and S7A). Of note, *TLO*γ*4* was scored as ‘hypofilamentous’ upon Tet induction relative to the uninduced condition because it was hyper-filamentous in the–Dox conditions relative to wildtype levels of filamentous growth in the presence of Dox. Induction of *TLO* expression on Spider media did not alter filamentous growth for any of the assayed strains (S7B Fig). Thus, Tet-regulated expression of *TLO* genes affected filamentous growth in a condition-dependent manner influenced by nutrient, carbon source, stress, and potentially other environmental conditions.

**Fig 5:**
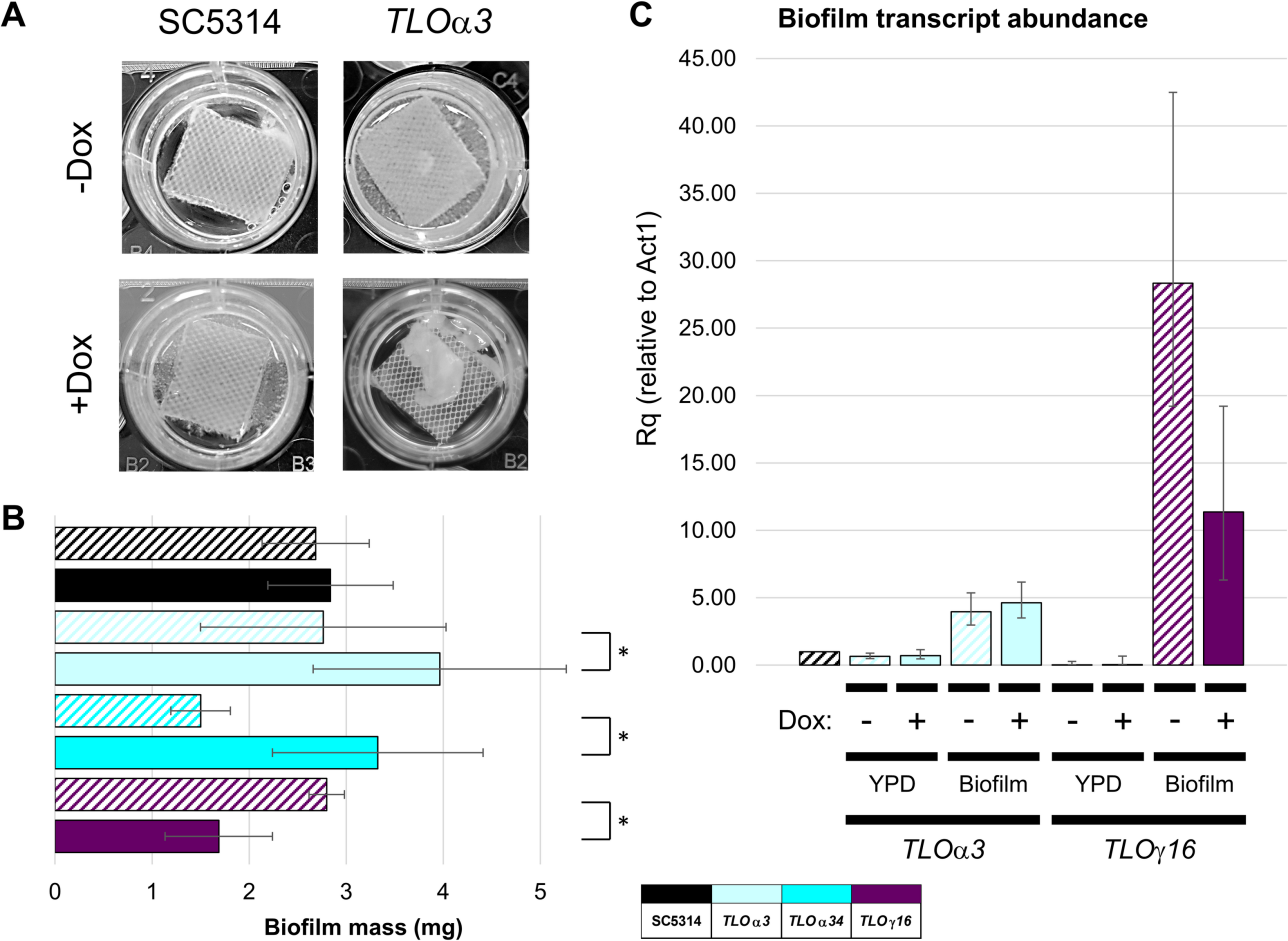
*TLO* induction impacts biofilm production. **A.** Cultures were grown overnight either in the presence or absence of 50 μg/ml Dox and incubated with adult bovine serum pre-treated silicone squares in 12 well tissue culture plates with Spider media. Cells were incubated at 37°C with shaking for 90 minutes. Silicon squares with adherent cells were then removed, rinsed, and placed in new Spider media and allowed to develop biofilms for 65 hours. **B.** Biofilm mass was dried and quantified for a minimum of four biological replicates. *TLO*s whose induction produced a significant change in biofilm mass were plotted along with the SC5314 control. A legend indicates the representative *TLO* gene for each color where solid bars indicate +Dox and hatched bars indicate–Dox. * denotes p < 0.05. **C.** RNA was harvested from wildtype and Tet-regulated *TLO* strains prepared as in **A** for biofilm formation. Transcript abundance of the regulated *TLO* was determined by qRT-PCR using *ACT1* as a reference gene.

In *C*. *albicans*, biofilms require both the adhesion of yeast cells to the substrate at the base of the biofilm and subsequent filamentous growth to form an interwoven hyphal mat that accounts for much of the biofilm biomass [77]. Biofilm formation on silicone implanted devices is clinically relevant because it can seed disseminated infection and complicate patient treatment [49, 78, 79]. To assess biofilm formation, we used a simplified *in vitro* system in which cells were incubated with silicone elastomer squares and allowed to form communities for approximately 3 days (Fig 5A). *TLO* expression was induced overnight, prior to incubation on the silicone substrate, and was discontinued during the process of biofilm formation. Tet-regulated induction of two *TLO* genes, *TLO*α*34* and *TLO*α*9*, reduced biofilm mass in the absence of induction compared to the parental SC5314 strain (S8A and S8B Fig). When induced in the presence of Dox, *TLO*α*3* and *TLO*α*34* increased biofilm mass while *TLO*γ*16* decreased biofilm mass significantly relative to SC5314 (S8A and S8C Fig). Three genes affected biofilm biomass when induced. *TLO*α*3* and *TLO*α*34* increased biofilm mass and *TLO*γ*16* reduced biofilm mass when induced (Fig 5B). Transcript levels of both *TLO*α*3* and *TLO*γ*16* in the Tet-regulated strain increased dramatically during biofilm production compared to growth in liquid YPD (Fig 5C). Dox-induction of *TLO* expression yielded a small increase in *TLO*α*3* transcript abundance and a sharp decrease in *TLO*γ*16* transcript abundance relative to their–Dox levels (p = 0.013), which mirrors the change in biofilm production following induction. Thus, integration of the Tet-inducible expression system at specific *TLOs* altered phenotypes independent of induction of *TLO* expression with Dox. Additionally, biofilm formation is a complex phenotype involving multiple processes; accordingly, *TLO*s involved in biofilm production did not completely overlap with those contributing to filamentation or cell-cell adhesion.

### *TLO* expression has little impact on azole resistance

**Fig 6:**
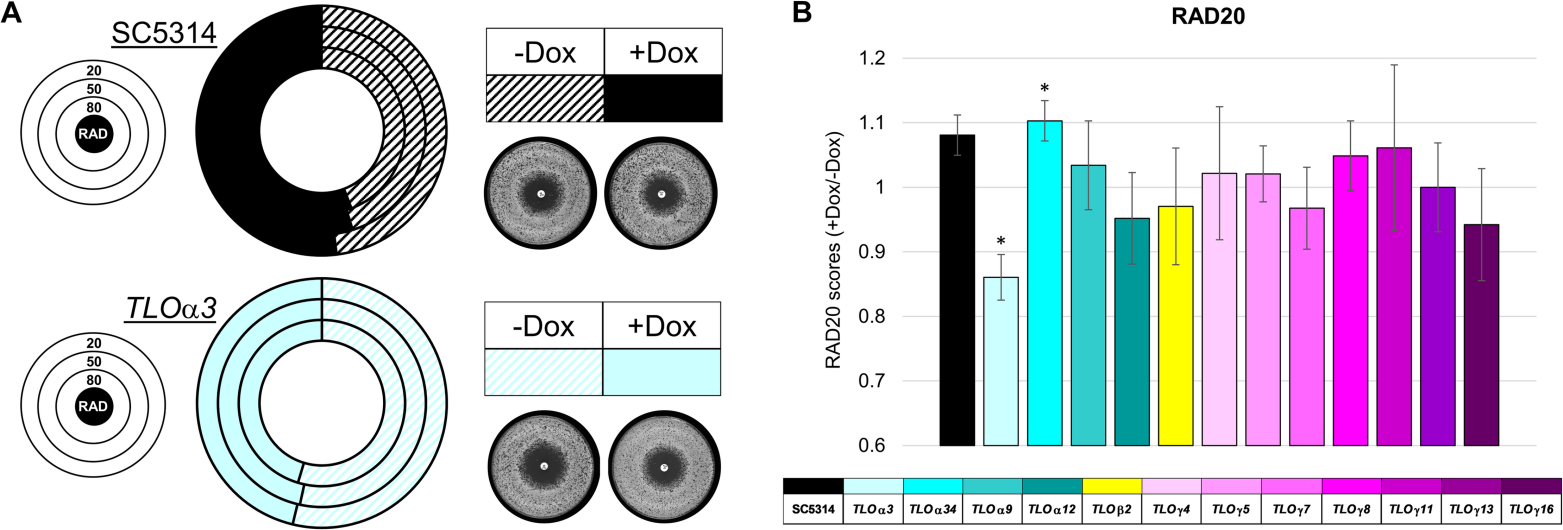
*TLO*s play a minor role in azole resistance. **A.** Tet-regulated *TLO* strains were grown overnight in the presence or absence of 50 μg/ml Dox. Cells were plated onto YPD and allowed to grow in the presence of a 25 μg fluconazole disc. Plates were photographed after 2 days. Analysis of RAD20, RAD50, and RAD80 is visualized using a doughnut plot. **B.** The average +Dox/-Dox RAD20 is plotted for all strains with standard deviation. A legend indicates the representative *TLO* gene for each color where solid bars indicate +Dox and hatched bars indicate–Dox. * denotes p < 0.05.

Recent work has highlighted a role for the Mediator tail subunit in resistance to azole class antifungal drugs [80–82], but involvement by *TLO* (Med2 in Mediator) was not specifically addressed. Tet-regulated *TLO* strains incubated overnight with or without Dox were plated onto solid agar and allowed to grow in the presence of a 25 μg fluconazole disc. After two days of growth, the size of the zone of inhibition appeared similar across most strains and induction conditions. The susceptibility phenotype (size of the zone of inhibition (ZOI)) of two induced *TLO*s, *TLO*α*3* and *TLO*α*34*, decreased and increased, respectively, when induced compared to the uninduced state (Fig 6). Changes in resistance due to regulated *TLO* expression were relatively minor, typically altering the size of the ZOI by no more than 15%. No alterations to fluconazole tolerance (measured by the fraction of growth inside the ZOI [83]) were apparent for any strain in the presence of absence of Dox and the rate of change in growth (slope) differed for only a single *TLO*, *TLO*γ*11*, in the presence of Dox (S9 Fig). Thus, expression of a few *TLO* genes, one telomeric and one located far from the telomeres had some effects on azole drug resistance, although this effect was neither broadly conserved among *TLO*s nor profound.

### *TLO*s play a significant role in virulence

**Fig 7:**
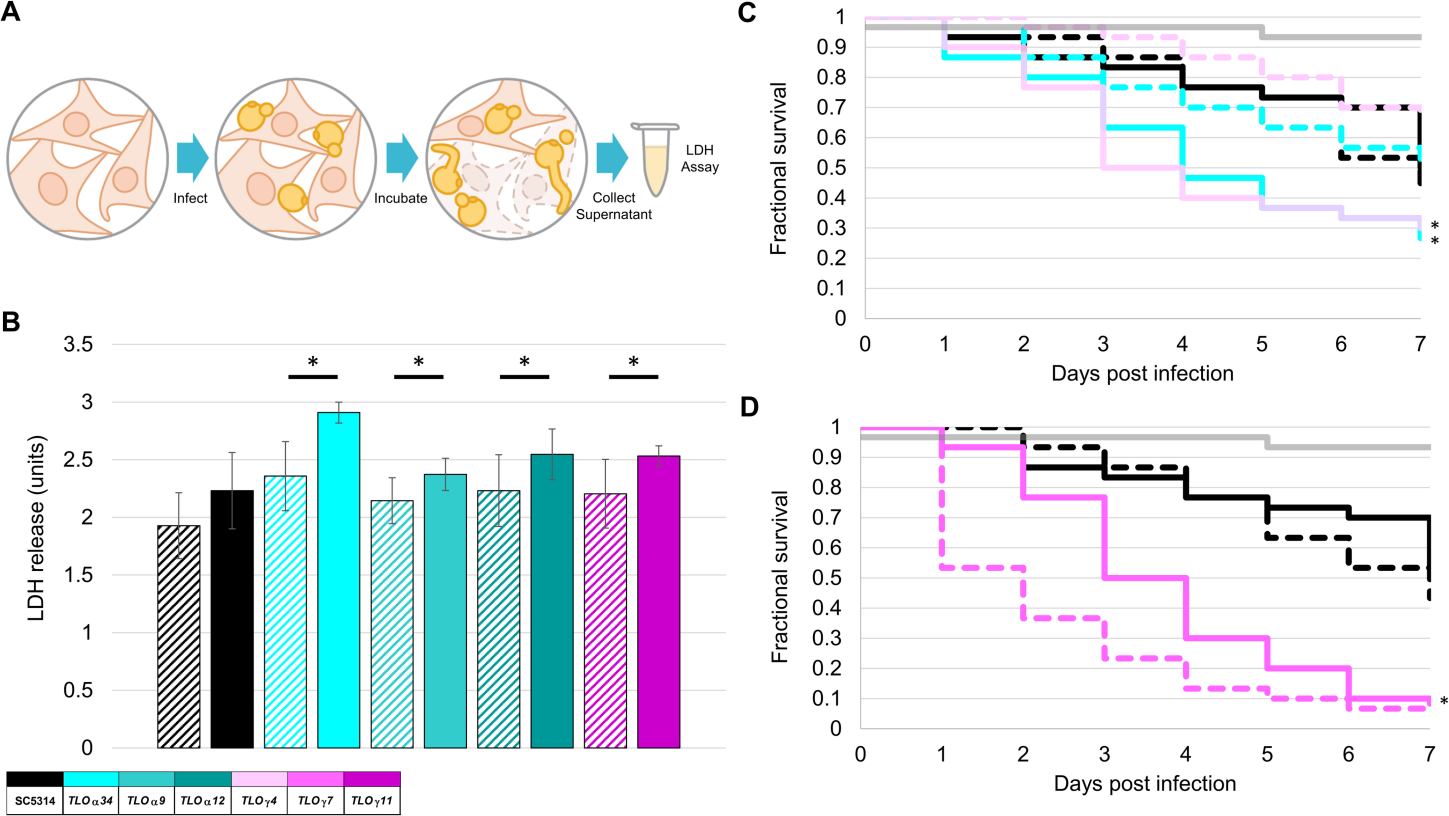
Virulence in macrophage and *Galleria mellonella* is regulated by multiple *TLO*s. **A.** *TLO* induction strains were grown overnight and then subcultured in log phase for 3 hours in YPD medium with or without 50 μg/ml Dox. Log phase *C*. *albicans* was inoculated onto macrophages at a MOI of 2 and incubated for 20 hours. Measurements of LDH release by macrophage quantified immune cell death. **B.** Induction of four *TLO*s significantly increased macrophage death. Data represents six biological replicates and standard deviation. *C*. *albicans* strains were injected into *G*. *mellonella* larvae and time to death determined. Experiments were performed using 10 larvae per strain (in triplicate). Two *TLO*s increased killing in the larvae (**C**) while one *TLO* exhibited decreased killing (**D**). A legend indicates the representative *TLO* gene for each color where solid bars indicate +Dox and hatched bars indicate -–Dox. * denotes p < 0.05.

To more directly test the role of *TLO*s in virulence, Tet-regulated *TLO* strains were co-incubated with RAW 264.7 macrophages *in vitro* at an MOI of two following logarithmic phase growth in the presence or absence of Dox. After 16 hours, LDH release from infected cultures was measured to quantify macrophage survival (Fig 7A). *C*. *albicans* cells with induced expression of *TLO*α*34*, *TLO*α*9*, *TLO*α*12*, or *TLO*γ*11* resulted in more macrophage death compared to the uninduced cells of the isogenic strain (Fig 7B).

To test virulence with an *in vivo* model, we infected *Galleria mellonella*, a model for disseminated candidiasis, with *C*. *albicans* [69]. Larvae were infected with overnight cultures of *C*. *albicans* cells that had been induced or not induced with Dox and larval survival was monitored during the infection. Induction of three *TLO*s, *TLO*α*34*, *TLO*γ*4 and TLO*γ*7*, altered the morbidity of infected *Galleria* worms. Of these, Tet-induced expression of two genes, *TLO*α*34* or *TLO*γ*4*, significantly increased lethality (Fig 7C), while induction of *TLO*γ7 reduced virulence compared to the uninduced state (Fig 7D). Thus, individual *TLO* genes, when induced, have different effects on virulence attributes such as macrophage lysis and *G*. *mellonella* viability.

### Phenotypic traits associate with individual *TLO*s and clades

**Fig 8:**
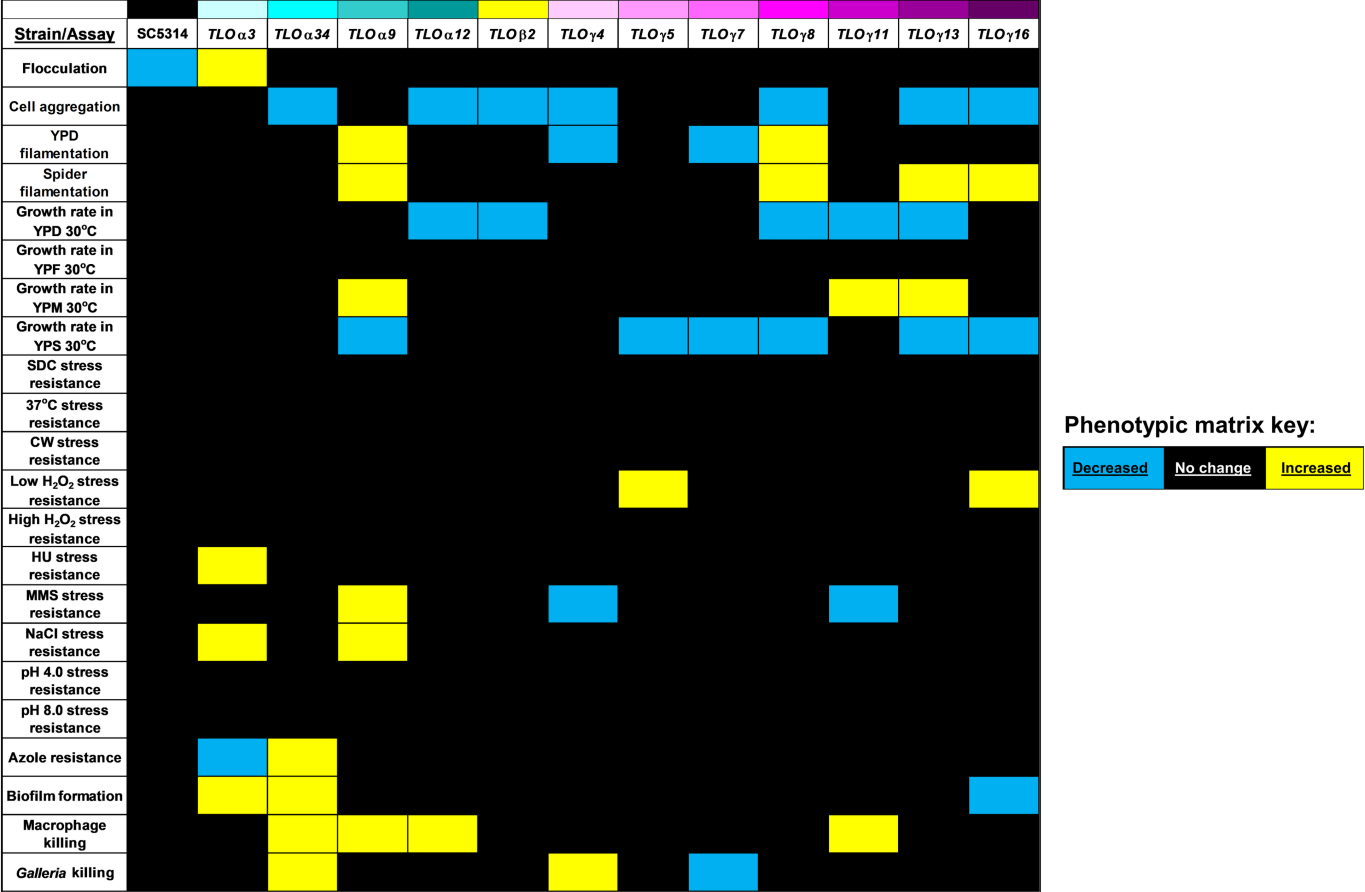
Phenotypic matrix of *TLO* functional diversity. Data for all phenotypic assays and investigated *TLO*s are summarized in a heat map where blue indicates a decrease in phenotype, black indicated no phenotype change, and yellow indicates an increase in phenotype.

Taken together, the above results reveal that *TLO* genes evolved varying degrees of influence on different virulence traits of *C*. *albicans*. A heat map displaying all significant associations of individual *TLO* expression (+Dox vs.–Dox) with each assayed phenotype reveals that there are few conserved functions shared by most of the *TLO* genes (Fig 8). Induced *TLO* expression promoted unidirectional changes in a number of phenotypes such as cell aggregation, growth at 30°C, and macrophage killing. Yet, a number of phenotypes can change in either direction upon induction of specific *TLO* genes. Thus, *TLO* gene family members have shared and unique modes of transcriptional regulation. This suggests a complex pattern of genotype-phenotype associations due to evolution and inheritance of *TLO* genes in *C*. *albicans*.

**Fig 9:**
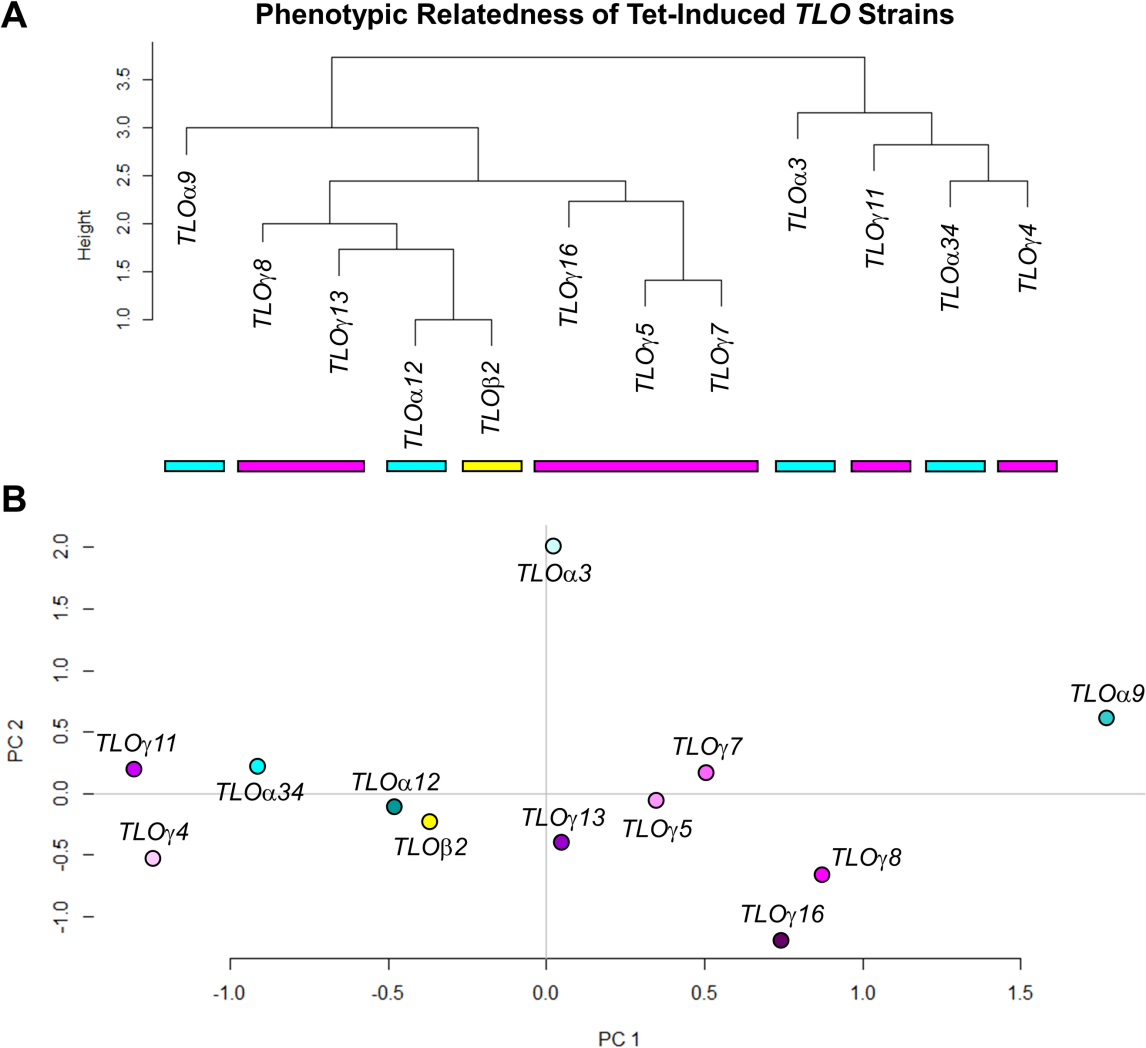
*TLO* relatedness does not correlate with phenotypic outcomes of induced expression. **A.** Changes in phenotype due to *TLO* induced expression were used to construct a dendrogram describing similarity in function. **B.** *TLO*s were plotted against principal components based on phenotypic data. PC1 and PC2 explains 25.5% and 19.6% of the data set variation, respectively. Two *TLO*γ gene clusters flank most of the *TLO*α genes and *TLO*β2.

To better visualize the relationship between *TLO* genes in controlling phenotypic traits, hierarchical clustering was performed using the phenotypic data for all Tet-induced loci. Comparison of phenotypic scores (Fig 8) in all pairwise combinations for the *TLO* genes served as the basis for calculated relative distance (S10 Fig). One major branch-point separated the *TLO*s into two main clusters, which were each composed of a mixture of *TLO*α and *TLO*γ genes (Fig 9A). This suggests that the functions acquired by different Tlo proteins are not clade-specific. Yet, replotting the data for each *TLO* using principal components analysis (PCA) assigned 25.5% and 19.6% of the variation among the data set to PC1 and PC2, respectively (Fig 9B). Interestingly, this approach separated the *TLO*γ genes into two clusters on either side of the main *TLO*α genes cluster and *TLO*β2. The two *TLO*γ groups separated primarily along PC1 with *TLO*γ4 and *TLO*γ11 being lower on PC1. A single gene, *TLO*α*9*, remained an outlier. This suggests that a mixture of clade-associated and *TLO*-specific features produce the functional variation observed among Tet-regulated *TLO* strains.

### Selective pressures map to specific sites within the *TLO*s

**Fig 10:**
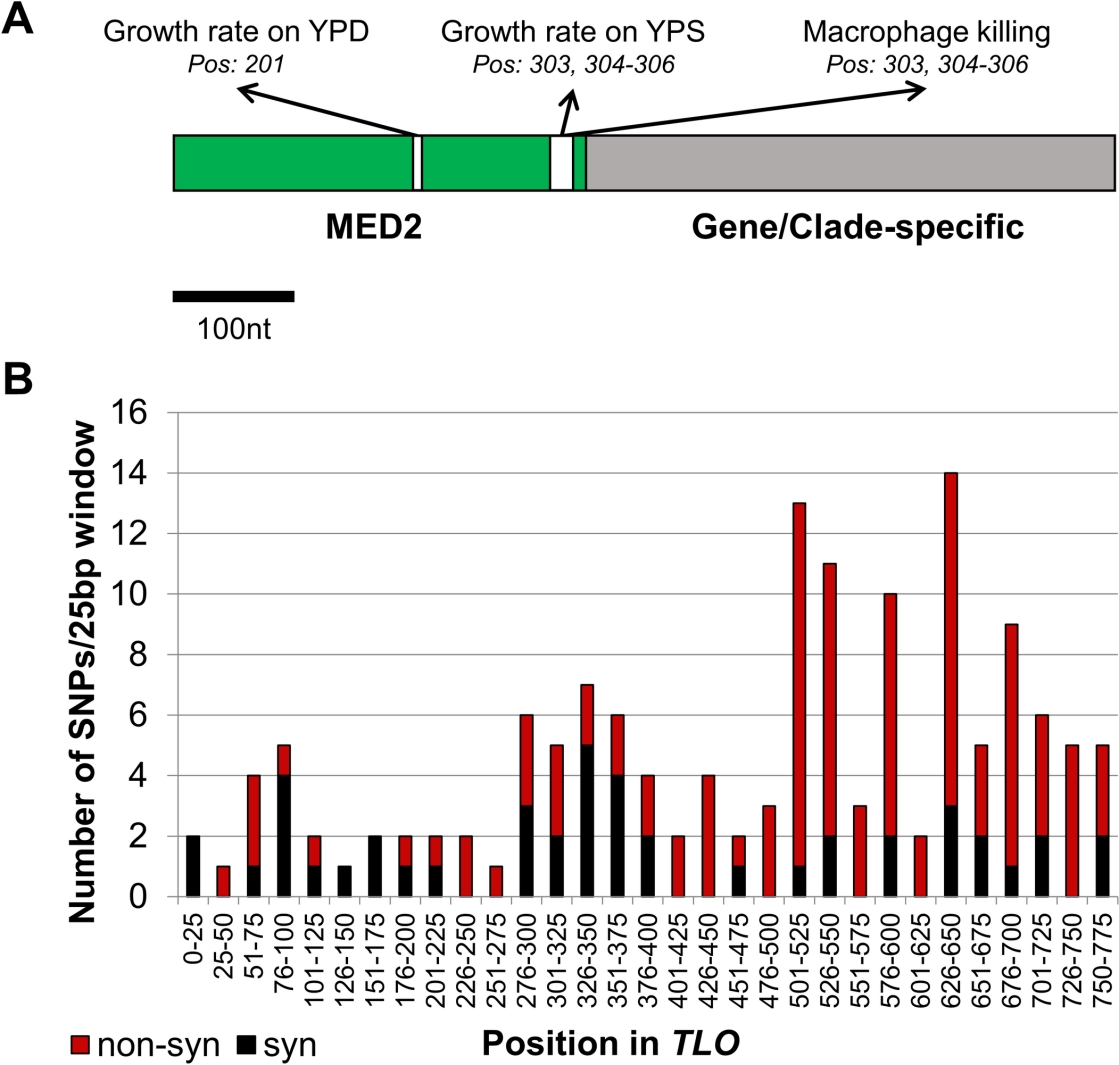
Different selective pressures have acted on the *TLO* gene sequence and can be assigned to specific positions. **A.** Correlation of phenotypic data to nucleotide positions across the Med2 domain identified three sites (shown in white) that significantly affect specific phenotypic assays. **B.** The mutational history of the *TLO*s was reconstructed using phylogenetic relatedness of the full gene sequence. The number of unique synonymous (black) and nonsynonymous (red) mutations are plotted in 25 basepair windows. Synonymous mutations dominated in the Med2 domain but were the minority of mutations within the gene/clade-specific region.

The sequence of *TLO* genes can be separated into roughly two halves, an N-terminal Med2 domain and a C-terminal gene/clade-specific region [64]. While the Med2 domain is responsible for the association of Tlo with the Mediator complex, the function of the C-terminal region is less clear and may interact with specific transcription factors to recruit RNA polymerase II through Mediator [65, 84]. To map individual phenotypes to specific polymorphisms that differ between members of the *TLO* gene family, we focused only on the Med2 domain, as the gene/clade specific region sequence diverged too much to allow individual substitutions to be analyzed across all *TLO* clades. Within the first 315 nucleotides (nt) of the genes, encompassing the Med2 domain, seven polymorphisms could be correlated relative to 15 phenotypes that were altered upon *TLO* induction. Three phenotypes mapped to specific sites within the Med2 domain. Doubling time in YPD rich media associated specifically with a synonymous polymorphism (A or G) at nucleotide 201 (p = 0.034) (Fig 10A). Two traits, the ability to lyse macrophages and the growth rate in YPS mapped to polymorphisms at nucleotide positions 303 to 306 near the end of the Med2 domain (p = 0.025). This polymorphic site includes synonymous G to A transition at position 303 together with a three nucleotide CGT indel beginning at position 304, which alters the coding sequence by introducing an arginine. Many other positions in the clade/gene specific region of *TLO*s may affect phenotypic properties of *C*. *albicans*, but the high prevalence of indels following the Med2 domain precludes a systematic analysis.

Comparing variants in *TLO* sequences to the phylogenetic tree allows a reconstruction of the mutational history of the *TLO* gene family during evolution. To identify mutations that arose during gene family expansion, two closely-related *TLO*s (i.e., *TLO*γ*5* and *TLO*γ*13*) were compared to build a common ancestral sequence that occupied the node connecting those two genes (S11A Fig). This process was repeated until all nodes were connected through reconstructed sequences. This reconstruction identified 146 unique mutations that arose during *TLO* expansion, with most polymorphisms clustered towards the 3’ end of the gene in the gene/clade-specific domain (S10B Fig). Importantly, the ratio of non-synonymous to synonymous mutations was highest in the gene/clade-specific region; the Med2 domain harbored a significantly higher frequency of synonymous than non-synonymous SNPs. This suggests that different evolutionary pressures are operating on the *TLO* sequence: purifying selection acting on infrequently maintained SNPs has promoted sequence identity of the Med2 domain, while positive selection has diversified the gene/clade-specific domain. This implies that all of the Tlo proteins continue to function through their interaction with Mediator.

Sequencing of *TLO*α*34*, the one *TLO* gene not located at a telomere, identified multiple polymorphisms relative to the genome reference sequence, including nine SNPs (four of them non-synonymous substitutions that produced significant amino acid changes (A502D, V509D, V511D, and L535S) and two insertions/deletions (indels) within the 3’ gene/clade-specific domain of *TLO*α*34* compared to the Assembly 21 (A21) sequence (S12 Fig., S3 Table). Together with an eighteen nucleotide insertion and three nucleotide deletion, these mutations suggest that rapid *TLO* evolution is not limited to those genes found within subtelomeres.

*TLO* sequences within a clade had relatively neutral selection coefficients (mean K_a_/K_s_ of 0.76 and 1.25 for *TLO*α and *TLO*γ intra-clade diversity, respectively), which increased dramatically to K_a_/K_s_ = 5.33 between *TLO*β*2* and *TLO*α-clade ancestral sequences and to K_a_/K_s_ = 2.45 between *TLO*α/β and the *TLO*γ ancestral sequences (S11A Fig). The average selection coefficient for all *TLO*s is higher than for other *Candida* expanded gene families, including the serine aspartyl proteases (SAPs) in C. *albicans* (K_a_/K_s_ = 1.70), the *EPA* adhesins in *C*. glabrata [85] (K_a_/K_s_ = 1.41), and other expanded gene families in *C*. *albicans* (S11B Fig). Thus, it appears that selection has propelled divergence within the *TLO* gene family that appears to have had phenotypic consequences on *TLO* function.

## Discussion

Evolutionary studies of functional divergence following gene duplication commonly analyze variation between two paralogous sequences, to facilitate direct comparison. The degree to which extensive gene family expansion associates with continued functional diversification remains largely unexplored due to the complex nature of assessing individual family members for specific phenotypes. Here, we performed functional analysis of 12 of the 14 *C*. *albicans TLO* genes and found that different *TLO*s regulate distinct phenotypic properties to different degrees. Each *TLO* affected multiple phenotypes and most phenotypes were affected by multiple *TLO* genes when individual *TLO* genes were induced for expression. The *TLO* gene family has undergone extensive genotypic evolution with a significant proportion of variation occurring within the gene/clade-specific 3’ end, which is experiencing significant positive selection for acquired mutations. Furthermore, phenotypic variation in three traits could be mapped to specific polymorphic sites in the *TLO* gene family, suggesting specific mutational events following gene duplication lead to diverse functions.

The *TLO* gene family encodes a highly similar set of interchangeable protein subunits, yet individual genes affect distinct sets of biological functions. For example, induction of *TLO*α*9* altered outcomes in seven phenotypic assays ranging across cell growth, filamentous growth, stress responses, and interactions with macrophages. Additionally, most *TLO* genes caused a mixture of phenotypic outcomes when induced, suggesting that a single *TLO* likely affects the differential expression of a significant number of downstream genes to produce the observed phenotype. Indeed, the two *C*. *dubliniensis TLO* homologs each regulate a large combination of unique and overlapping gene sets that promote exclusive and overlapping phenotypes [86]. We assume that incorporation of a particular Tlo protein into the Mediator complex may shift the relative expression of a distinct set of genes and thereby modulate a particular phenotypic response. The resulting phenotypic plasticity has the potential to confer a repertoire of available Mediator ‘types’ that could operate as a primary driver of *TLO* expansion. Thus, retention of divergent *TLO*s would act as a bet-hedging mechanism by which shifts in the incorporation of certain Tlo proteins would provide greater adaptability during changes in growth conditions or new host niches.

Induced expression of individual *TLO*s provided the most direct route to assess paralog function. Regulated transcription of candidate genes can overcome difficulties in phenotypic expression due to compensation and redundancy but introduces its own caveats such as toxicity, pathway overload, stoichiometric imbalance, and promiscuous interactions with non-physiological targets when a gene is overexpressed [87]. Thus, overexpression can produce phenotypes that are not directly attributable to the gene of interest but other affected cellular processes [88–90]. Indeed, genetic analysis using an inducible deletion or overexpression system in *C*. *albicans* found disagreements between the two approaches that may reflect these effects [91]. Furthermore, the strength of induced expression in *C*. *albicans* can alter observed phenotypes [92, 93]. Aberrant phenotypes produced by induced *TLO* expression were mitigated, in part, by a lack of noticeable toxicity and association with known pathways. Previous studies demonstrated that Tlo proteins exist in excess of other Mediator components as a free Tlo pool [65, 94], which suggests inherent stoichiometric imbalance with regards to Mediator. The Tlo incorporated into Mediator also appears quite plastic as multiple Tlos have been biochemically purified from the complex [65]. It is possible that induced *TLO* expression leads to target promiscuity either through Mediator’s role in transcriptional regulation or the Mediator tail’s role in chromatin remodeling [95]. However, the excess of Tlo protein found in *C*. *albicans* favors a model in which induced expression alters the relative availability of the regulated Tlo to be incorporated into Mediator and regulate expression of gene sets through interaction with different transcription factors although target promiscuity may occur.

Most tested phenotypes were affected by multiple different *TLO* genes. Nearly all *TLO*s had a similar effect on cell aggregation; in contrast, regulation of growth in YPS and macrophage killing fell primarily on specific *TLO* clades. In most cases, at least two separate *TLO* genes affected each phenotype altered by *TLO* induction and different subsets of *TLO* genes modulated most of the phenotypes (Fig 8). Furthermore, different genes that regulated the same phenotype displayed both enhancing and suppressing effects, indicating that the evolution of individual *TLO* genes as well as *TLO* clades likely influences the phenotypic consequences of induced expression.

15 of 22 phenotype assays detected a phenotype associated with induction of at least one or two of the 12 *TLO* genes tested. Six of the seven phenotypes that were not affected corresponded to different environmental stresses such as pH and high temperature, and other stresses typically were significantly affected by only one or two Tet-induced *TLO* genes, suggesting that the *TLO* genes may not have a prominent role in stress responses. Previous expression profiling of SC5314 grown in a range of stress conditions that overlap with those tested here (pH 4.0, pH 8.0, Calcofluor white, etc.) supports this hypothesis: *TLO* genes did not display significant expression changes in a range of stress conditions [96]. This contrasts sharply with Med2 and other tail components of Mediator in *S*. *cerevisiae* that have integral roles in the regulation of general stress response pathways [97, 98]. On the other hand, overlapping contributions to a given phenotype among *TLO* genes may reduce the phenotypic effect of inducing a single gene or result in minor effects that are difficult to distinguish. Therefore, contributions of individual *TLO*s to some phenotypes may be underscored or missed entirely.

A notable exception is exposure to MMS, which methylates DNA and leads to DNA replication fork stalling. Mutants of the single copy of *MED2* in *S*. *cerevisiae* display defects in viability following DNA damage analogous to the results here seen with induction of certain *TLO*s (*TLO*α*9*, *TLO*γ*4*, and *TLO*γ*11*) [99, 100]. We suggest that this may reflect a broader role for *MED2* and Mediator in DNA regulation and repair rather than transcriptional regulation in response to extracellular stress.

In contrast to the role in stress response, induction of a diverse set of *TLO* genes affected growth rates in a range of carbon sources but did not significantly alter growth in minimal media. In some cases, the same gene (i.e., *TLO*α*9*) produced opposite effects when grown in media supplemented with two different disaccharide sugars. The *C*. *albicans* genome encodes 20 different hexose transporters that are regulated through complex signaling networks that remain to be fully elucidated [101, 102]. Modulation of these pathways by *TLO* genes could have important phenotypic consequences on carbon source utilization, which in turn could have major effects on *C*. *albicans* biology and host interactions [103–105]. Of note, induction of *TLO* gene expression altered growth rates either by unidirectionally increasing or decreasing doubling times for any single carbon source. Similarity in the metabolic response to induced expression of disparate *TLO* genes across clades suggests a conserved role for Med2 subunits that may have existed prior to *TLO* expansion.

Induction of *TLO* expression also had a profound impact on filamentous growth and biofilm formation in different media contexts. Different *TLO* genes promoted or suppressed filamentous growth and biofilm formation although the *TLO*s involved varied depending on the conditions used. Thus, *TLO*s whose induction increased filamentous growth did not promote biofilm formation and vice versa. The critical step of cell-cell adhesion decreased when most *TLO*s were induced, although correspondence with decreased biofilm mass was only seen for Tet-induced expression of *TLO*γ*16*. Interestingly, expression of *TLO*γ*16* decreased following induction and biofilm formation whereas *TLO*α*3* increased in expression and biofilm formation after induction, suggesting individual *TLO*s may affect different components of the regulatory circuits controlling biofilm formation that can, in turn, affect their own expression [106]. Thus, the cumulative steps of cell adhesion and filamentous growth are not necessarily additive for biofilm formation. Since biofilm formation can proceed without induction of the filamentous growth transcriptional circuit and filamentous growth does not necessarily yield biofilms, the two processes are not entirely codependent [77, 107, 108]. Modulation via expression of different Tlo proteins may play a direct role in promoting different aspects of biofilm formation, including adhesion, filamentous growth, and substrate invasion. Indeed, Mediator components have fundamental roles in cell state transitions that are analogous to yeast-hyphal differentiation in other organisms [109–111], suggesting a conserved role for the complex in defining cell type-specific expression. In *C*. *albicans*, Mediator and the tail module, which includes Med3 and Med2/Tlo, function within the phenotypic switch between the ‘sterile’ white and mating-competent opaque states [66]. Thus, *TLO*s have the potential to control cell state transitions broadly across the breadth of cell types described for *C*. *albicans* [55, 112–114] and, more specifically, for adhesion and the yeast-hyphal transition important for flocculation and biofilm formation.

Induced expression of single *TLO* genes had surprising effects on complex phenotypes such as immune cell survival and virulence. Unexpectedly, Tet-induced expression of a single member of the large paralogous *TLO* gene family increased the ability of *C*. *albicans* to kill macrophages and *G*. *mellonella* hosts. While many *C*. *albicans* mutants have defects in virulence (reviewed in [53]), attributing a given mutation to virulence traits can be complicated by general fitness defects. In contrast, *TLO*s that regulated pathogenicity had equivalent or slightly reduced fitness when induced despite also displaying increased virulence. This suggests that *TLO*s modulate genes specific to virulence properties that do not significantly influence growth and filamentous growth processes under the assayed conditions *in vitro*.

The subtelomeric position of the *TLO* genes exposes them to significantly elevated frequencies of expression variability and genome change relative to other regions of the genome. Variability in *TLO* expression is observed in these strains consistent with previous reports although this expression plasticity is somewhat dampened [115], presumably due loss in variation of the regulated allele. The observed variation in expression may act to promote variation in available Mediator ‘types’ by altering the available Tlo pool over time [115]. This could explain some of the phenotypic variability in these assays, as the relative abundance of the regulated Tlo in the total cellular pool is being altered but is still competing with other Tlos for incorporation into Mediator to produce an observable effect. The subtelomeric context of *TLO*s may also account for, in part, the large number of polymorphisms that distinguish each *TLO* gene among its paralogous sequences. Yet *TLO*α*34*, the only non-telomeric *TLO* gene, underwent significant sequence evolution as well, indicating an underlying chromosomal or genetic feature that contributes to this process. Additionally, it is reasonable to assume selection has acted on the *TLO* gene family during expansion. Extensive sequence variation exists among *TLO* family members and there is strong evidence of positive selection during their evolution, especially at the major nodes that separate the three *TLO* clades. Thus, a large number of SNPs differentiate the *TLO* clades and these SNPs often produces a change to the protein sequence. Interestingly, different selective pressures appear to be operating across the *TLO* gene sequence. Purifying selection across the Med2 domain likely reflects the continued requirement for integration within Mediator [116], whereas positive selection operates on the variable 3’ end of the gene that has evidence of encoding a transcriptional activation domain (TAD) [94, 116]. Variation within the TAD could provide a mechanism for recruitment of different transcription factors. Emergence of SNPs within *TLO* sequences to produce allelic variants may further differentiate function within a single gene although we have identified few heterozygous SNPs within chromosomal homologs of single *TLO* genes. This may be a consequence of frequent recombination between chromosome homologs in the subtelomeres that rapidly fixes heterozygous positions through gene conversion or break-induced replication [33, 36].

Importantly, an indel position at the end of the Med2 domain was associated with growth in sucrose and macrophage interaction, demonstrating that variant positions may be under selection for specific phenotypes. Numerous indels within the gene/clade-specific region complicates further analysis of variant positions in the 3’ end of the *TLO*s. Yet, it is tempting to speculate that divergent evolution of the *TLO* sequence, especially within the TAD, affects phenotypic plasticity among the Tet-regulated strains by affecting the expression of different sets of target genes. Thus, expansion of the Med2-domain containing *TLO*s in *C*. *albicans* led to sequence variation that results in phenotypic variation to promote a highly adaptive lifestyle.

## Materials and methods

### Strains and growth conditions

Strains of *Candida albicans* used in this study are listed in S1 Table. Strains were grown on YPD agar at 30°C unless otherwise noted. For induction of the tetracycline-inducible system, cultures were grown overnight in 3 mL of YPD liquid media with constant agitation in the presence (induced) or absence (uninduced) of 50 μg/mL of doxycycline. Saturated cultures were then prepared for individual experiments using their respective protocols.

### Transformant construction

Strains were transformed by standard lithium acetate transformation procedures as described previously through multiple rounds of transformation [117]. For integration of the tetracycline-inducible system at the endogenous *TLO* locus, the tetracycline-responsive promoter, the reverse tetracycline transactivator (rtTA), and the nourseothricin resistance marker *(SAT1*) were amplified from plasmid pNIM6 [70] using primers ALO110 and ALO111. Primer sequences are listed in S2 Table. These primers target this amplicon to the native *TLO* locus corresponding to a direct integration at the ATG start codon. The integration site was determined by polymerase chain reaction (PCR) using primers ALO108 and ALO109, corresponding to the *p*_*TET*_ promoter and downstream in the *TLO* coding sequence, respectively. In some cases, additional sequencing was required to specify the *TLO* targeted by integration. Amplification of the integration site with ALO108 and primers ALO2*2*5, ALO226, and ALO227, which bind farther downstream within the clade-specific region, were used identify integration at specific *TLO* genes for clades α, γ, and β, respectively.

### Quantitative real-time (qRT-PCR) analysis

RNA was collected from 2x10^6^ cells grown for four hours in liquid YPD medium in the presence or absence of 50 μg/mL of doxycycline. Cells were removed from the medium and RNA isolated using the MasterPure Yeast RNA Purification Kit (EpiCentre, Madison, WI) according to the manufacturer’s instructions. Subsequently, 1 μg of RNA was used to synthesize cDNA using oligo-(dT)_18_ and Superscript III reverse transcriptase (Thermo Scientific, Waltham, MA). cDNA was assayed for genomic DNA contamination using intron-spanning primers, ALO30 and ALO31, for ribosomal protein large subunit 6 (*RPL6)* and only cDNA lacking genomic contamination was used for qRT-PCR (S4 Table).

qRT-PCR was performed with PowerUp SYBR Green (Applied Biosystems, Foster City, CA) using an Applied Biosystems QuantStudio 3 qPCR machine and analyzed with the QuantStudio Design and Analysis Software package version 1.4.2. Primers used are listed in S4 Table. Quantification of individual *TLO* genes was assessed relative to *ACT1*. The comparative Rq method was used measure expression levels. Experiments for each gene were performed a minimum of three biological replicates in technical duplicates.

### Growth rate analysis

Overnight cultures were grown in 300 μL YPD liquid medium with or without 50 μg/mL doxycycline. Cultures were diluted 1:2000 into the appropriate growth medium with continued application or lack thereof of doxycycline. Optical density was measured every 15 minutes for 18–48 hours at 30°C shaking at 250rpm using an AccuSkan FC plate reader (Fisher Scientific, Hampton, NH). The polynomial measurement of the curve was used to derive doubling times. These experiments were completed with a minimum of three biological replicates with two technical replicates each.

### Flocculation assay

Overnight cultures were grown in YPD liquid medium in the presence or absence of 50 μg/mL of doxycycline. Cultures were vortexed and diluted to an initial optical density (OD_600_) of 2.0 in a channeled cuvette. OD_600_ readings were taken at the start of the assay and every 15 minutes on a ThermoFisher NanoDrop One (Fisher Scientific, Hampton, NH) for a total of 210 minutes to plot cell settling. These experiments were completed with six biological replicates.

### Cell aggregation

Overnight cultures were grown in YPD liquid medium in the presence or absence of 50 μg/mL of doxycycline. Cultures were diluted 1:2 in a total volume of 100 μL YPD liquid. An aliquot was visualized across a minimum of 10 random fields of view using a Leica DM750 with an attached Leica MC170HD digital camera (Leica, Wetzlar, Germany). The number of cells per aggregate was tallied across all fields of view and plotted as average for each induced *TLO* gene with standard error. Two biological replicates at a minimum were performed per strain.

### Filamentation assay

Overnight cultures were grown in YPD liquid medium in the presence or absence of 50 μg/mL of doxycycline. Cells from each overnight culture were counted by hemocytometer and plated at a concentration of 100 cells per plate onto solid YPD and Spider medium. These plates were grown at 30°C for 7 days and imaged using a BioRad ChemiDoc XRS+ imaging system (BioRad, Hercules, CA). Images were processed by the visual analysis tool MIPAR v1.4.1 (MIPAR, Worthington, OH) and scored using the following formula: Filamentation score = 100 * (C_f_) * (0.8 (R_h_/R_y_) + 0.2 (S_w_)). C_f_ is the proportion of filamenting cells, R_h_ is the radius of the hyphal halo, R_y_ is the radius of the yeast colony, and S_w_ is the score for colony wrinkling. Three biological replicates were performed at a minimum per strain.

### Adhesion and invasion assay

Overnight cultures were grown in YPD liquid medium in the presence or absence of 50 μg/mL of doxycycline. Cells were counted with a hemocytometer and plated at a concentration of 100 cells per plate onto solid YPD and Spider medium. These plates were grown at 30°C for 5 days and imaged prior to rinsing as described above for filamentation. A steady stream of water was run over the plate to remove non-adherent colonies and imaged. Remaining colonies were then rubbed off with a gloved finger and imaged to assess agar invasion. Three biological replicates were performed at a minimum per strain.

### *In vitro* biofilm growth and biomass determination

Biofilm production and measurement was performed as outlined in (Nobile et al, *Cell*, 2012). Briefly, silicone squares were pre-treated overnight in 12 well tissue culture plates with 2 mL of adult bovine serum. Wells were washed with PBS and 2 mL of Spider medium was added to each well. Overnight cultures were grown in YPD liquid medium in the presence or absence of 50 μg/mL of doxycycline. Cells were introduced at an OD_600_ of 0.5 to each well and incubated for 90 minutes at 37°C, shaking at 120 rpm. Silicone squares were then removed with sterile forceps, rinsed in a separate PBS wash well, and transferred to a new well with 2 mL of Spider media. Cultures, now adhered to the silicone squares, were incubated for 60–65 hours at 37°C, shaking at 120 rpm. After incubation, media was gently pipetted from the wells and plate was left to dry on benchtop, slightly ajar, for 24 hours. Produced biofilm was then scraped off and weighed. Four biological replicates were performed at a minimum per strain.

### Environmental stress survival

Overnight cultures were grown in YPD liquid medium at 30°C in the presence or absence of 50 μg/mL of doxycycline. Cell density was determined using OD_600_ and cultures were adjusted to an OD_600_ of 1.0 in 1 mL ddH_2_0. These dilutions were used as a base for five sequential ten-fold dilution done in a 96 well plate. Or each stress condition, 5 μl of each dilution was spotted to the appropriate prewarmed agar plates including a synthetic complete defined (SCD) medium plate absent any stressor as a control for growth. Plates were then incubated at 30°C unless otherwise indicated and imaged at 24 hours and 48 hours.

### Macrophage LDH release assay

*C*. *albicans* macrophage killing was assessed by using the CytoTox96 nonradioactive cytotoxicity assay (Promega, Madison, WI). RAW 264.7 macrophages were seeded at 2.5 x 10^4^ cells per well in a 96 well plate in RPMI supplemented with 10% fetal bovine serum (FBS) and incubated overnight at 37°C and 5% CO_2._ Overnight *C*. *albicans* cultures were grown in YPD liquid medium at 30°C in the presence or absence of 50 μg/mL of doxycycline. These overnight cultures were then diluted 1:20 and grown for 3 hours into logarithmic phase growth in YPD medium with or without DOX. Log phase *C*. *albicans* cultures were then washed with PBS three times, inoculated into macrophages at a multiplicity of infection (MOI) of 2, and incubated overnight at 37°C and 5% CO_2_. To assess macrophage killing, plates containing *C*. *albicans* infected macrophage were centrifuged at 250 x g for 5 minutes and 10 μL from each well was transferred to a new plate. The transferred solution was diluted 1:5 with 40 μL of PBS and assayed using the Promega CytoTox Assay Kit according to the manufacturer’s instructions. The abundance of lactate dehydrogenase (LDH) release was calculated according to the manufacturer’s protocol.

### Galleria mellonella virulence assay

*Galleria mellonella* infections were carried out using previously described protocols (Fuchs et al. 2010). In brief, overnight cultures were grown in YPD liquid medium at 30°C in the presence or absence of 50 μg/mL of doxycycline. Cultures were washed 3 times in 5 ml sterile PBS. Cell density was quantified through hemocytometer. Cell suspensions (~2.5x10^5 CFUs) in a 10 μl volume of sterile PBS were injected into the terminal pro-leg of *G*. *mellonella* larvae (Vanderhorst Wholesale, www.snackworms.com) using a 26 G, 10 μl syringe (Hamilton, No.80300) (n = 30 larvae per *TLO*). Dilutions of cell suspensions were plated onto YPD agar and CFUs counted to confirm inoculum. After infection, *G*. *mellonella* larvae were incubated at 37°C for 7 days. *G*. *mellonella* larvae were scored daily for signs of death (immobility and darkened pigmentation). The Log-rank (Mantel-Cox) test was used for statistical analysis of survival curves.

### Fluconazole disk diffusion assay

Overnight cultures were grown in YPD liquid medium at 30°C in the presence or absence of 50 μg/mL of doxycycline. Cells for each strain were cultured overnight in YPD at 30°C in the presence or absence of 50 μg/mL of doxycycline. Optical density measurements were used to dilute the cultures to 0.04 OD/ml (800,000 cells/ml) and 70 μL plated onto solid YPD agar. Inoculated plates were left for one hour to dry and a single 25 μg fluconazole disc (Liofilchem, TE, Italy) was placed in the center of the plate. Cells were allowed to grow for 48 hours at 30°C and images taken using a BioRad ChemiDoc XRS+ imaging system (BioRad, Hercules, CA). Drug resistance was quantified using the diskImageR program which allows for analysis of drug response parameters [83].

### Hierarchical clustering

Alignment of *TLO* sequences was performed using the Multiple Sequence Comparison by Log Expectations (MUSCLE) [118]. A phylogenetic reconstruction was produced using maximum likelihood in MEGA7.

Phenotypic correlations between TLOs were produced by converting significant phenotypic changes across all assays into either a 1, 0, or -1, indicating an increased, unchanged, or decreased phenotype, respectively. A dendrogram was constructed from this matrix using Euclidean distances in R (v3.4.2) [119]. Principal components were constructed and visualized using the pca3D package.

### Statistical analysis

Statistics were performed using Microsoft Excel or R (v3.4.2) developed by the R Development Team [119]. Statistics were performed with a Student’s t-test unless otherwise annotated.

## Supporting information

S1 Fig *TLO* transcript abundance in the absence or presence of Dox. Tet-regulated *TLO* strains were grown for 4 hours in the presence or absence of 50 μg/ml Dox and transcript abundance of each regulated *TLO* was determined by qRT-PCR using *ACT1* as a reference gene.
(TIF)

S2 Fig Growth rates across a range of nutrient conditions are affected by induced *TLO* expression. Tet-regulated *TLO* strains were grown overnight in the presence or absence of 50 mg/ml Dox. Cells were diluted 1:2000 and grown in logarithmic phase for 16 hours at 30°C under sustained +/–Dox conditions. Growth on different minimal media, YP without added sugar (**A**), Spider media (**B**), sorbitol (**C**), and glycerol (**D**). A minimum of three replicates was performed for each condition. A legend indicates the representative *TLO* gene for each color. * denotes p < 0.05.
(TIF)

S3 Fig *TLO*s do not influence response to a variety of stressors. Cells were grown overnight in the presence or absence of 50 μg/ml Dox and plated using ten-fold spot dilutions starting at an OD_600_ of 1.0 on SCD solid agar media in the absence of Dox. Growth at 30°C (**A**), 37°C (**B**), 6mM H_2_O_2_ (**C**), pH 4.0 (**D**), pH 8.0 (**E**), and 100 μg Calcofluor white (**F**) was unaffected by *TLO* induction. A minimum of two replicates was performed for each condition.
(TIF)

S4 Fig *TLO*α*3* alters HU resistance at high concentrations. **A**. Cells were grown overnight in the presence or absence of 50 μg/ml Dox and plated using ten-fold spot dilutions starting at an OD_600_ of 1.0 on SCD solid agar media containing 2mM HU in the absence of Dox. **B**. Cells were grown overnight in the presence or absence of 50 μg/ml Dox and diluted 1:2000 into 96-well plates containing 10-fold dilution of HU ranging from 2M to 200μM. Cells were grown in logarithmic phase for 16 hours at 30°C and the doubling time calculated using a polynomial best fit line. Three replicates were performed for each strain and condition. * denotes p < 0.05.
(TIF)

S5 Fig Cell size was not affected by *TLO* induction. Strains were grown overnight in the presence or absence of 50 μg/ml Dox were diluted 1:2 and visualized by light microscopy. Bar = 30 μm.
(TIF)

S6 Fig Induced expression of most *TLO*s does not alter agar adhesion and invasion. Tet-regulated *TLO* strains were grown for 5 days on YPD and Spider solid agar plates following induction in the presence or absence of 50 mg/ml Dox. Prior to testing for adhesion, colony morphology was imaged (top). Water was then lightly run over the surface of the colonies to rinse off non-adherent colonies and imaged (middle). The top of the plate was rubbed lightly with a gloved finger under running water to visualize agar invasion and imaged (bottom).
(TIF)

S7 Fig *TLO* induction regulates condition-specific filamentation. Surface filamentation was imaged following 7 days of growth on YPD (**A**) and Spider (**B**) media at 30°C and quantified as in Fig 4B. A minimum of three replicates was performed for each data point. A legend indicates the representative *TLO* gene for each color where solid bars indicate +Dox and hatched bars indicate–Dox. * denotes p < 0.05.
(TIF)

S8 Fig Regulation of *TLO* integration and induced expression impacts biofilm formation. Biofilm production was assayed as described in Fig 5A. (**A**) Integration of the *p*_*TET*_ promoter significantly reduced biofilm formation of two *TLO*s, *TLO*α*34 and TLO*α*9*. (**B**). Induced expression of Tet-regulated *TLO*s significantly increased biofilm formation for two *TLO*s, *TLO*α*3 and TLO*α*34*, and reduced biofilm production in *TLO*γ*16*. These data are plotted together in (**C**). Data represents a minimum of four experiments. A legend indicates the representative *TLO* gene for each color where solid bars indicate +Dox and hatched bars indicate–Dox. * denotes p < 0.05.
(TIF)

S9 Fig Induction of *TLO* expression has little effect on tolerance or rate of change in growth to fluconazole. Tet-induced *TLO* strains were grown overnight in the presence or absence of 50 μg/ml Dox. Cells were plated onto YPD and allowed to grow in the presence of a 25 μg fluconazole disc. Plates were photographed after 2 days. The tolerance as measured by FOG20 (**A**) and rate of change of growth across the plate as measured by “slope” (**B**) were not affected by induced *TLO* expression, with the exception of slope for *TLO*γ*11*. Data represents a minimum of three experiments. A legend indicates the representative *TLO* gene for each color where solid bars indicate +Dox and hatched bars indicate–Dox. * denotes p < 0.05.
(TIF)

S10 Fig Correlation of phenotypic scores across induced *TLO*s. Pairwise correlations of the phenotypic consequences following *TLO* induction were calculated for all genes. A heat map denotes similarity where yellow-red indicates positive correlations and green-cyan indicates negative correlations.
(TIF)

S11 Fig *TLOs* have undergone extensive positive selection. **A.** Selection coefficients (K_a_/K_s_) were calculated for all branch points within the *TLO* phylogeny. Major branch points separating the *TLO* clades (circled) possess exceptionally high K_a_/K_s_ values whereas intra-clade branch points have more neutral selection coefficients. **B.** Selection coefficients were determined across expanded *C*. *albicans* gene families (*SAP*, allantoate permease, vacuolar membrane, *TLO*) and *C*. *glabrata* (*EPA*) for all nodes within their respective phylogenies. All gene families show evidence of positive selection with *TLO*s exhibiting the greatest effect of selection.
(TIF)

S12 Fig Mutations have accumulated in the chromosome internal *TLO*𝛂*34* gene. **A.** Polymorphic sites identified between the Sanger sequenced *TLO*α*34* sequence used in this study and Assembly 21 (A21) are plotted across the gene. SNPs are highlighted in yellow and indels are highlighted in red. **B.** The resulting protein sequences from A21, both homologs in A22, and our Sanger sequenced *TLO***𝛂***34* are aligned for comparison. Stars indicate identical positions and dashes indicate indels.
(TIF)

S1 Table *C*. *albicans* strains used in this study. (TIF)

S2 Table Transcript abundance of Tet-induced *TLO* strains. (TIF)

S3 Table Polymorphisms among *C*. *albicans TLO*𝛂*34* sequences. (TIF)

S4 Table Primers used in this study. (TIF)
