# Supplementary figures and images for: Functional diversification accompanies gene family expansion of MED2 homologs in Candida albicans
Source: PLoS Genet. 2018 Apr 9;14(4):e1007326. doi: 10.1371/journal.pgen.1007326 (PMC5908203; doi:10.1371/journal.pgen.1007326)

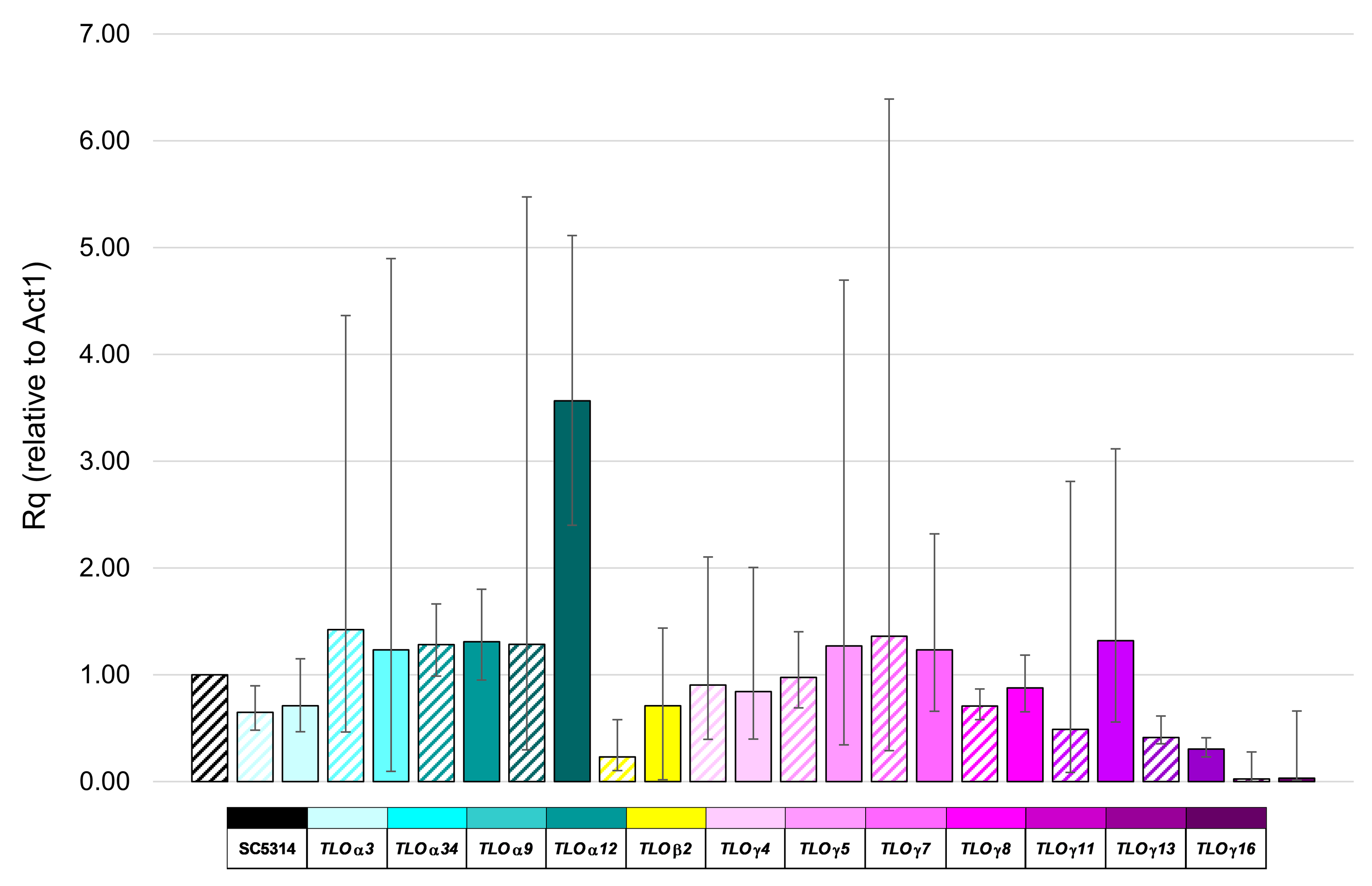

Supplement: S1 Fig — Tet-regulated TLO strains were grown for 4 hours in the presence or absence of 50 μg/ml Dox and transcript abundance of each regulated TLO was determined by qRT-PCR using ACT1 as a reference gene. (TIF) [file pgen.1007326.s001.tif]

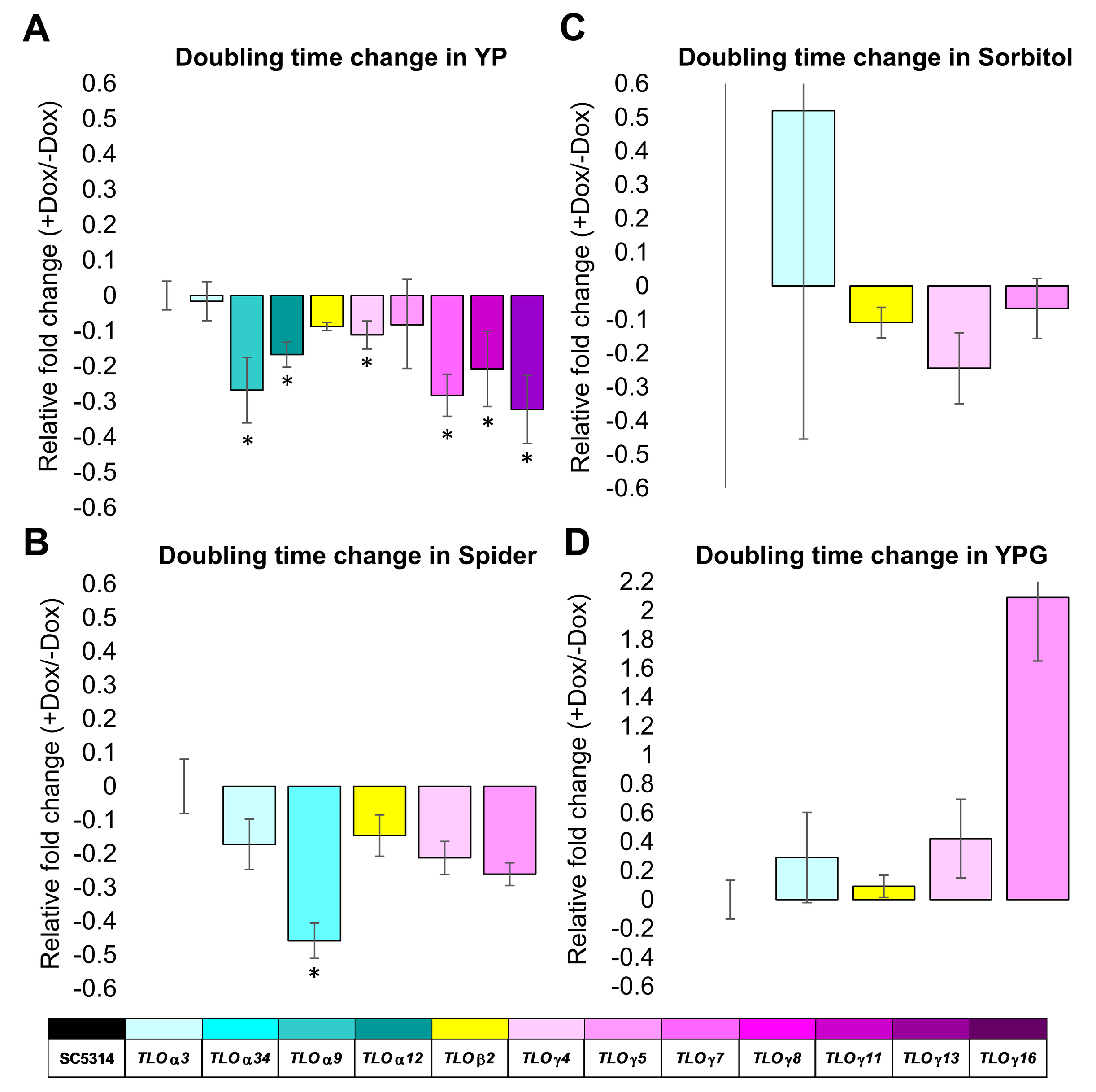

Supplement: S2 Fig — Tet-regulated TLO strains were grown overnight in the presence or absence of 50 mg/ml Dox. Cells were diluted 1:2000 and grown in logarithmic phase for 16 hours at 30°C under sustained +/–Dox conditions. Growth on different minimal media, YP without added sugar (A), Spider media (B), sorbitol (C), and glycerol (D). A minimum of three replicates was performed for each condition. A legend indicates the representative TLO gene for each color. * denotes p < 0.05. (TIF) [file pgen.1007326.s002.tif]

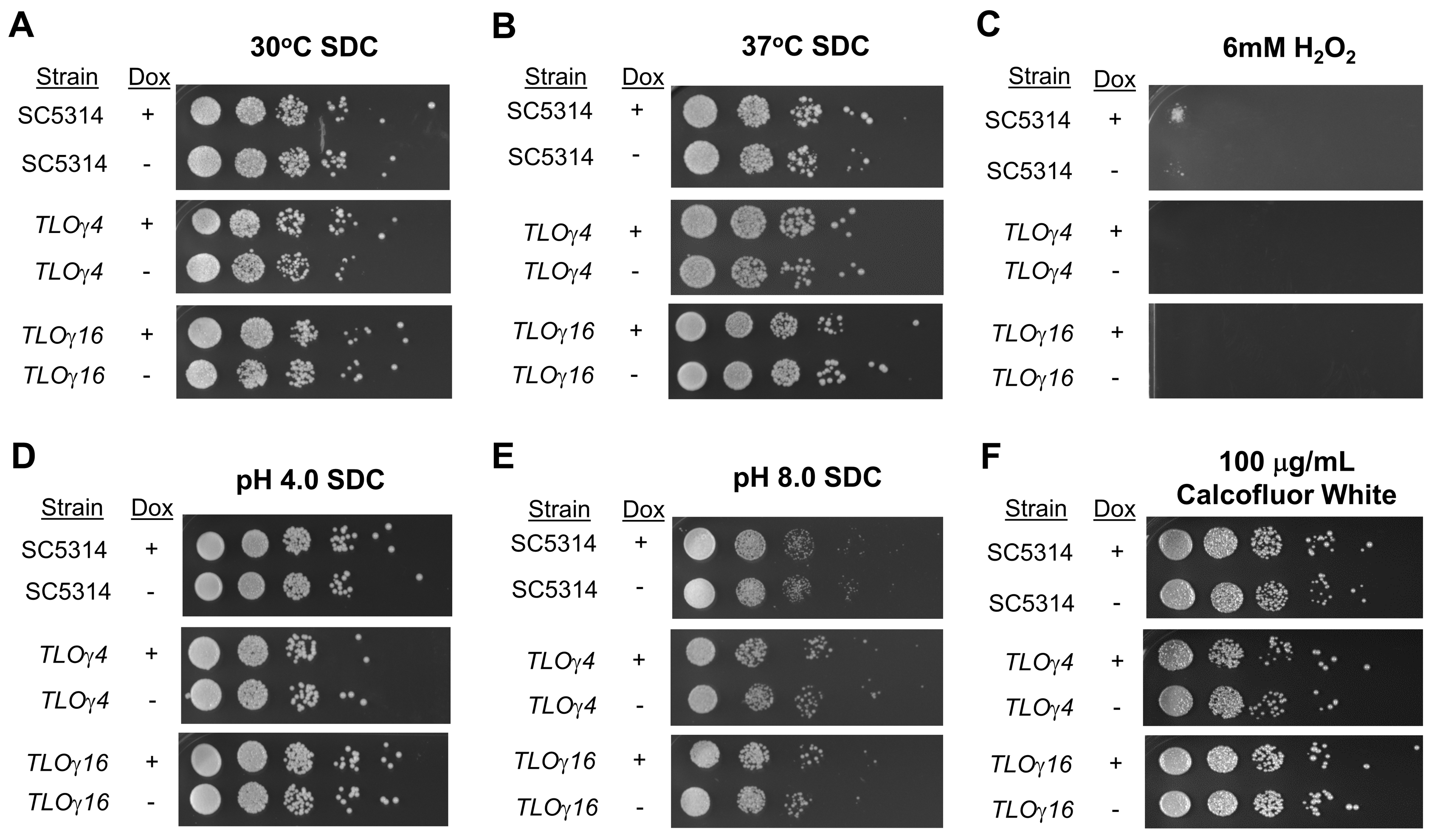

Supplement: S3 Fig — Cells were grown overnight in the presence or absence of 50 μg/ml Dox and plated using ten-fold spot dilutions starting at an OD600 of 1.0 on SCD solid agar media in the absence of Dox. Growth at 30°C (A), 37°C (B), 6mM H2O2 (C), pH 4.0 (D), pH 8.0 (E), and 100 μg Calcofluor white (F) was unaffected by TLO induction. A minimum of two replicates was performed for each condition. (TIF) [file pgen.1007326.s003.tif]

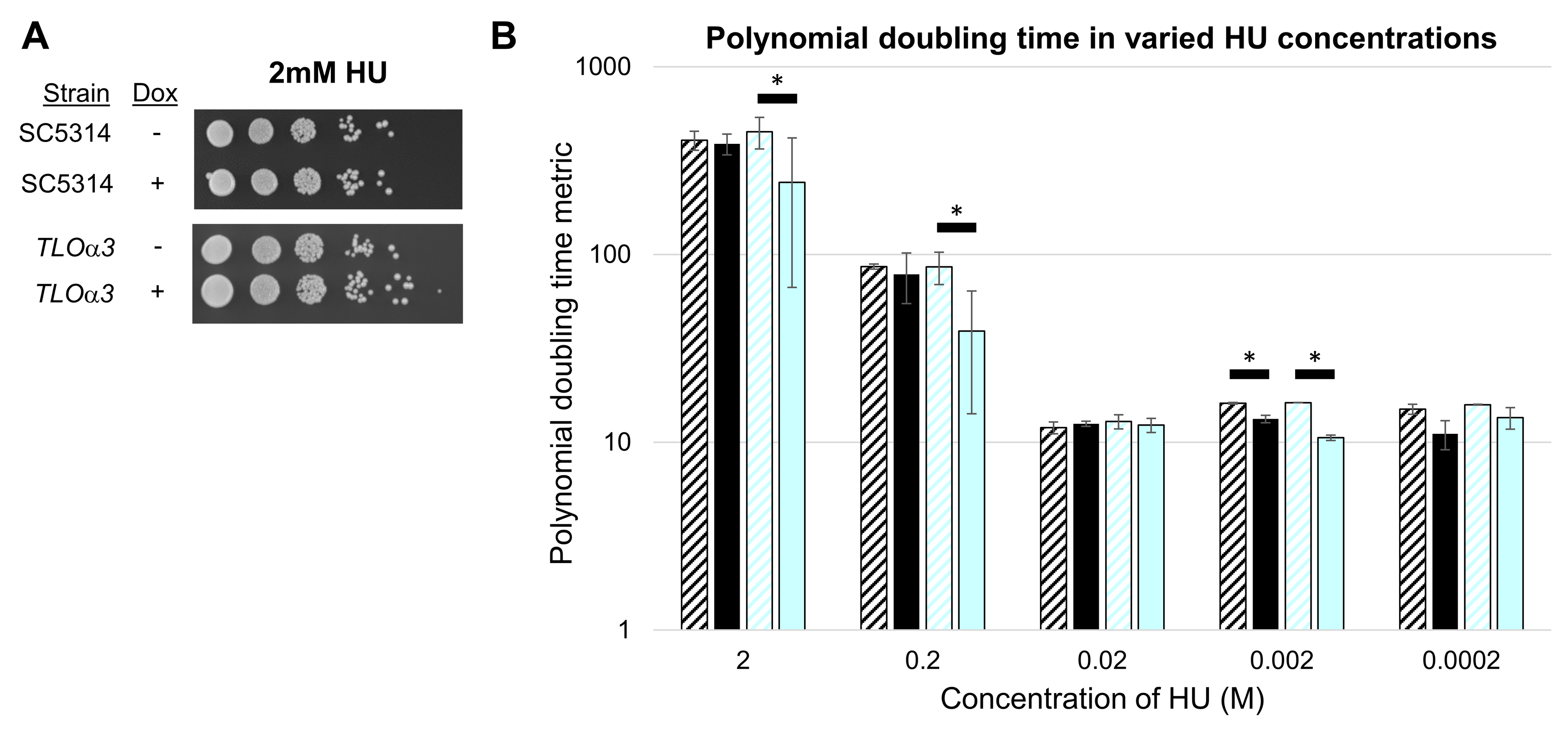

Supplement: S4 Fig — A. Cells were grown overnight in the presence or absence of 50 μg/ml Dox and plated using ten-fold spot dilutions starting at an OD600 of 1.0 on SCD solid agar media containing 2mM HU in the absence of Dox. B. Cells were grown overnight in the presence or absence of 50 μg/ml Dox and diluted 1:2000 into 96-well plates containing 10-fold dilution of HU ranging from 2M to 200μM. Cells were grown in logarithmic phase for 16 hours at 30°C and the doubling time calculated using a polynomial best fit line. Three replicates were performed for each strain and condition. * denotes p < 0.05. (TIF) [file pgen.1007326.s004.tif]

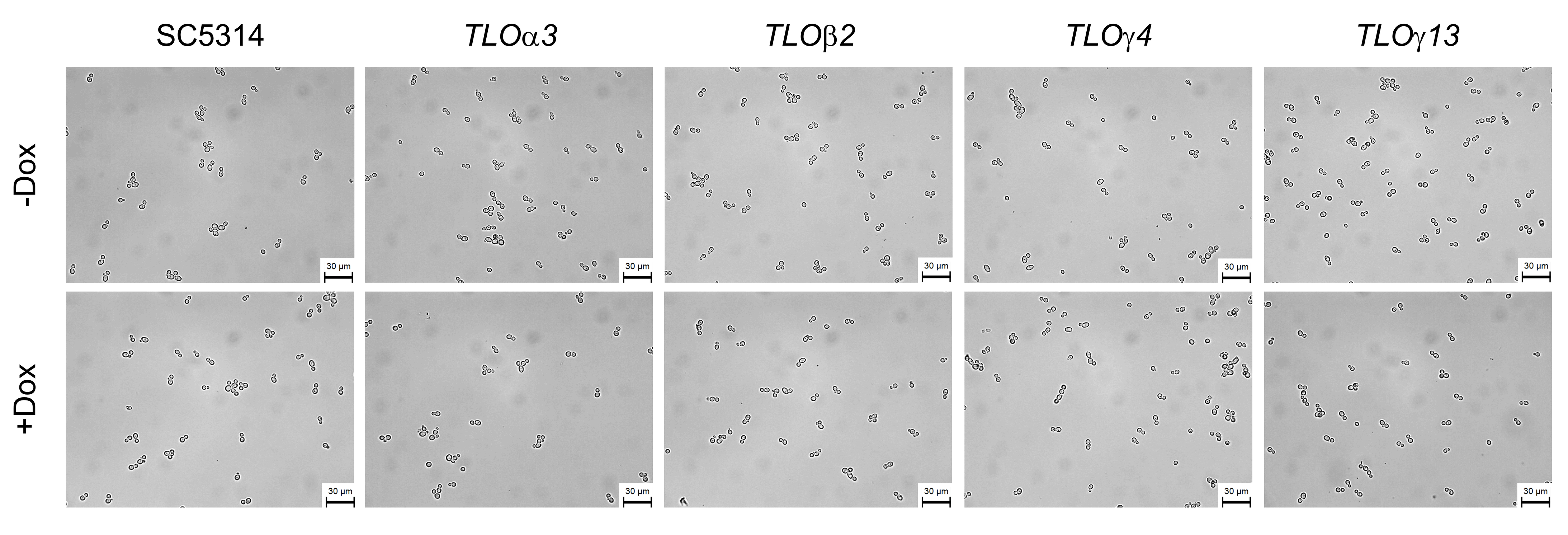

Supplement: S5 Fig — Strains were grown overnight in the presence or absence of 50 μg/ml Dox were diluted 1:2 and visualized by light microscopy. Bar = 30 μm. (TIF) [file pgen.1007326.s005.tif]

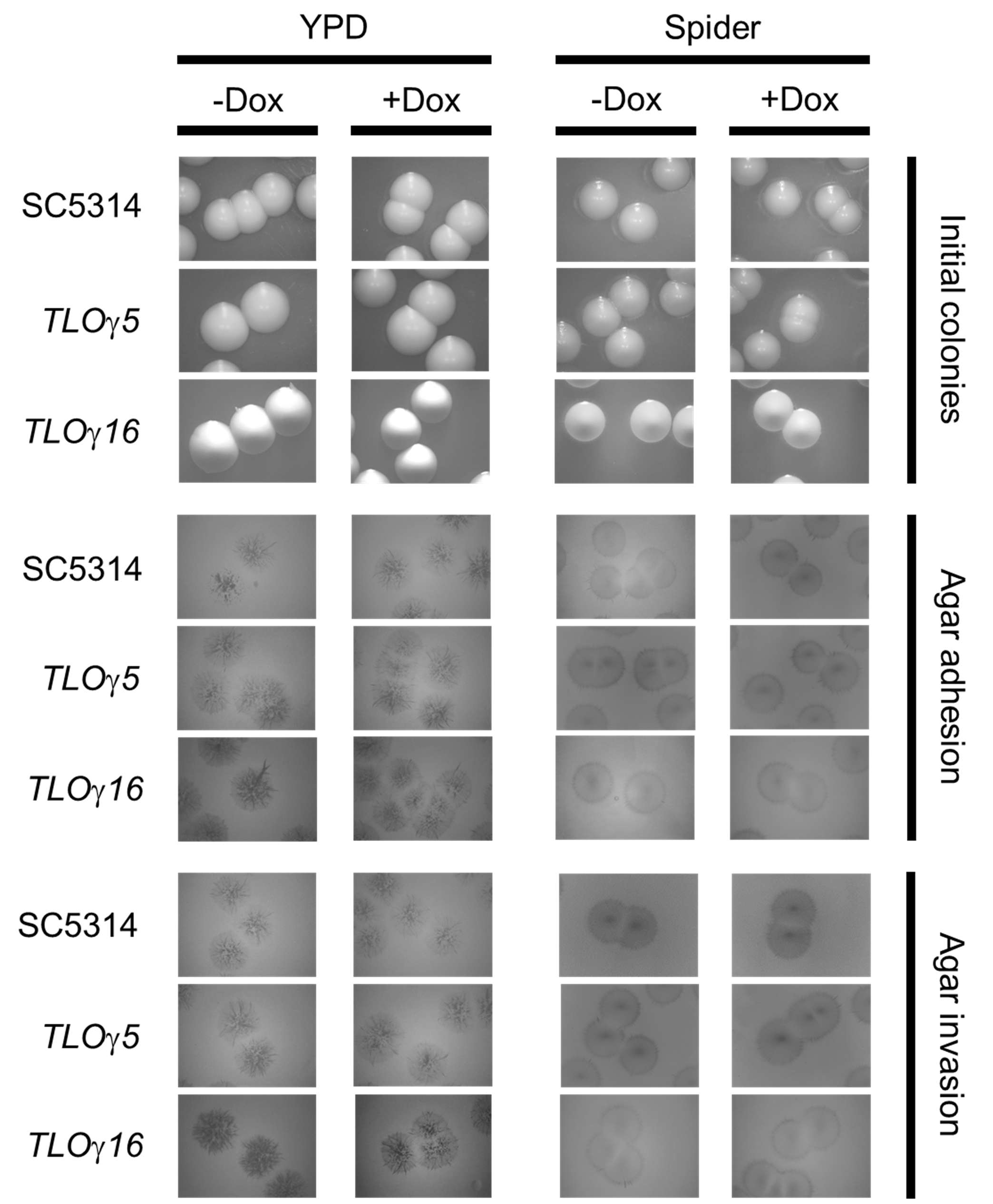

Supplement: S6 Fig — Tet-regulated TLO strains were grown for 5 days on YPD and Spider solid agar plates following induction in the presence or absence of 50 mg/ml Dox. Prior to testing for adhesion, colony morphology was imaged (top). Water was then lightly run over the surface of the colonies to rinse off non-adherent colonies and imaged (middle). The top of the plate was rubbed lightly with a gloved finger under running water to visualize agar invasion and imaged (bottom). (TIF) [file pgen.1007326.s006.tif]

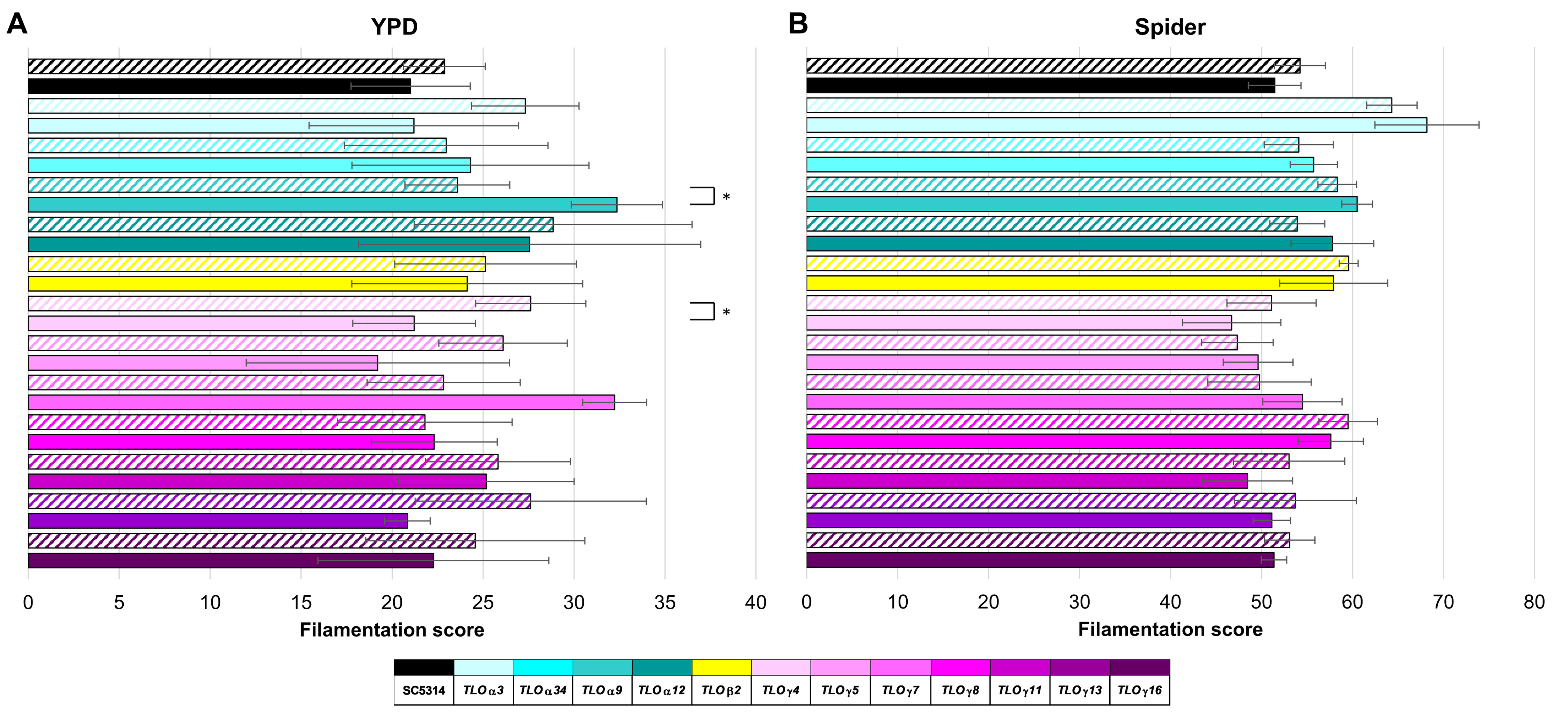

Supplement: S7 Fig — Surface filamentation was imaged following 7 days of growth on YPD (A) and Spider (B) media at 30°C and quantified as in Fig 4B. A minimum of three replicates was performed for each data point. A legend indicates the representative TLO gene for each color where solid bars indicate +Dox and hatched bars indicate–Dox. * denotes p < 0.05. (TIF) [file pgen.1007326.s007.tif]

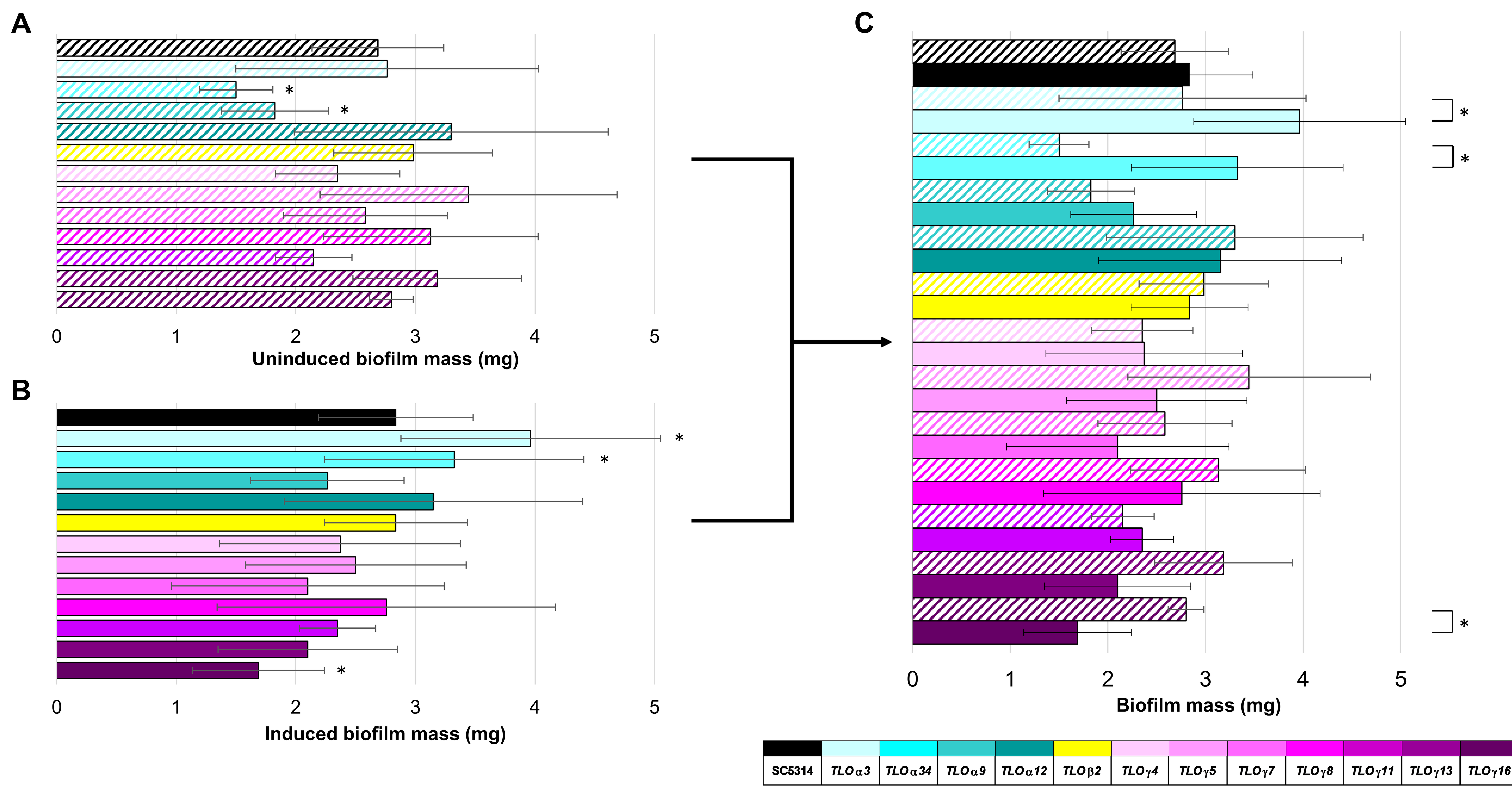

Supplement: S8 Fig — Biofilm production was assayed as described in Fig 5A. (A) Integration of the pTET promoter significantly reduced biofilm formation of two TLOs, TLOα34 and TLOα9. (B). Induced expression of Tet-regulated TLOs significantly increased biofilm formation for two TLOs, TLOα3 and TLOα34, and reduced biofilm production in TLOγ16. These data are plotted together in (C). Data represents a minimum of four experiments. A legend indicates the representative TLO gene for each color where solid bars indicate +Dox and hatched bars indicate–Dox. * denotes p < 0.05. (TIF) [file pgen.1007326.s008.tif]

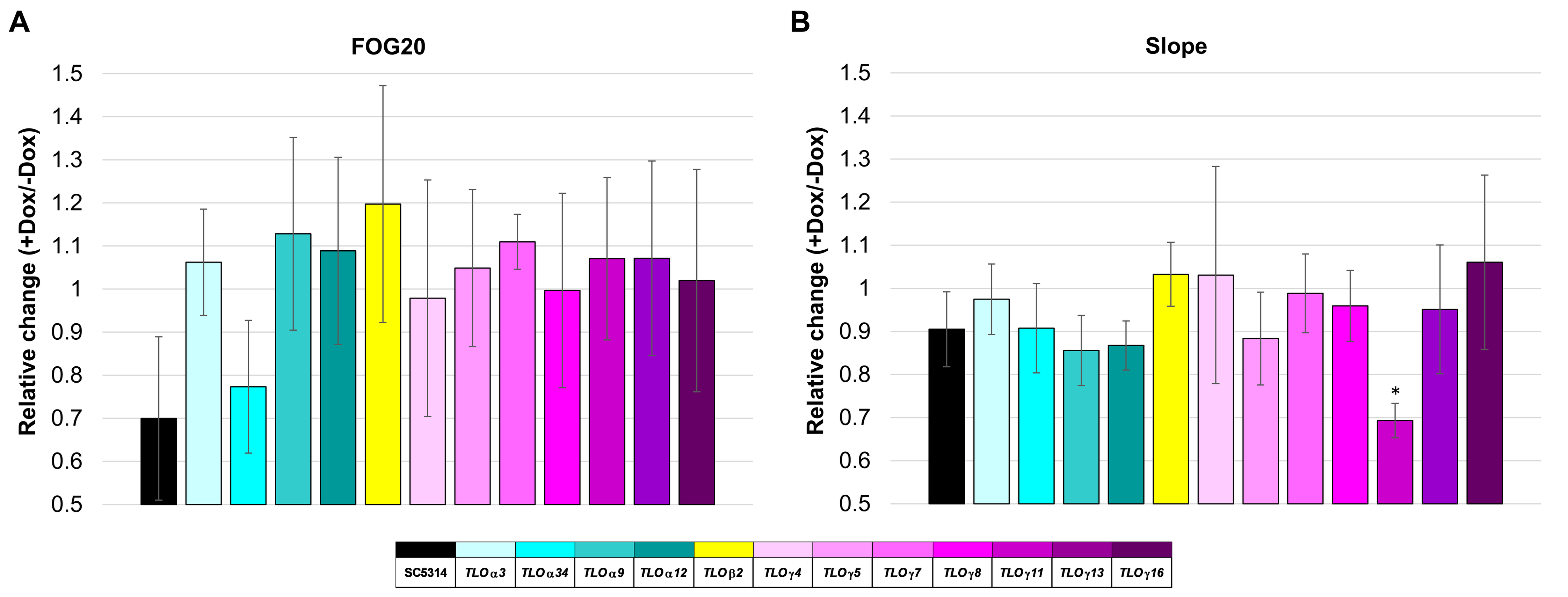

Supplement: S9 Fig — Tet-induced TLO strains were grown overnight in the presence or absence of 50 μg/ml Dox. Cells were plated onto YPD and allowed to grow in the presence of a 25 μg fluconazole disc. Plates were photographed after 2 days. The tolerance as measured by FOG20 (A) and rate of change of growth across the plate as measured by “slope” (B) were not affected by induced TLO expression, with the exception of slope for TLOγ11. Data represents a minimum of three experiments. A legend indicates the representative TLO gene for each color where solid bars indicate +Dox and hatched bars indicate–Dox. * denotes p < 0.05. (TIF) [file pgen.1007326.s009.tif]

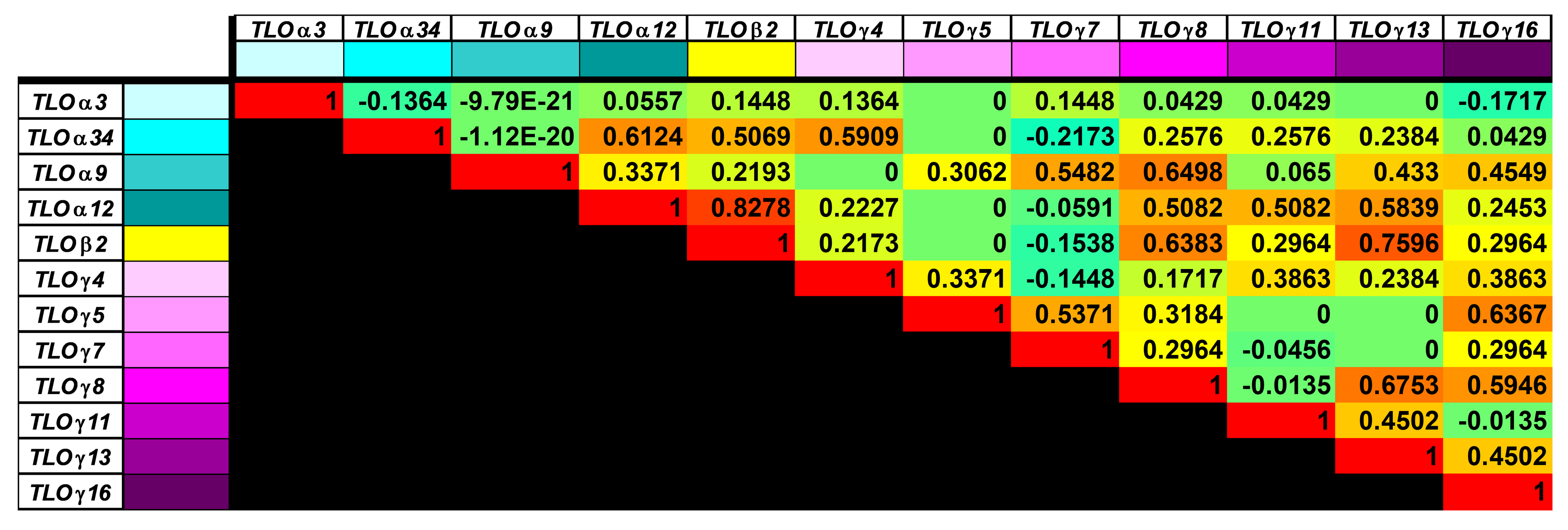

Supplement: S10 Fig — Pairwise correlations of the phenotypic consequences following TLO induction were calculated for all genes. A heat map denotes similarity where yellow-red indicates positive correlations and green-cyan indicates negative correlations. (TIF) [file pgen.1007326.s010.tif]

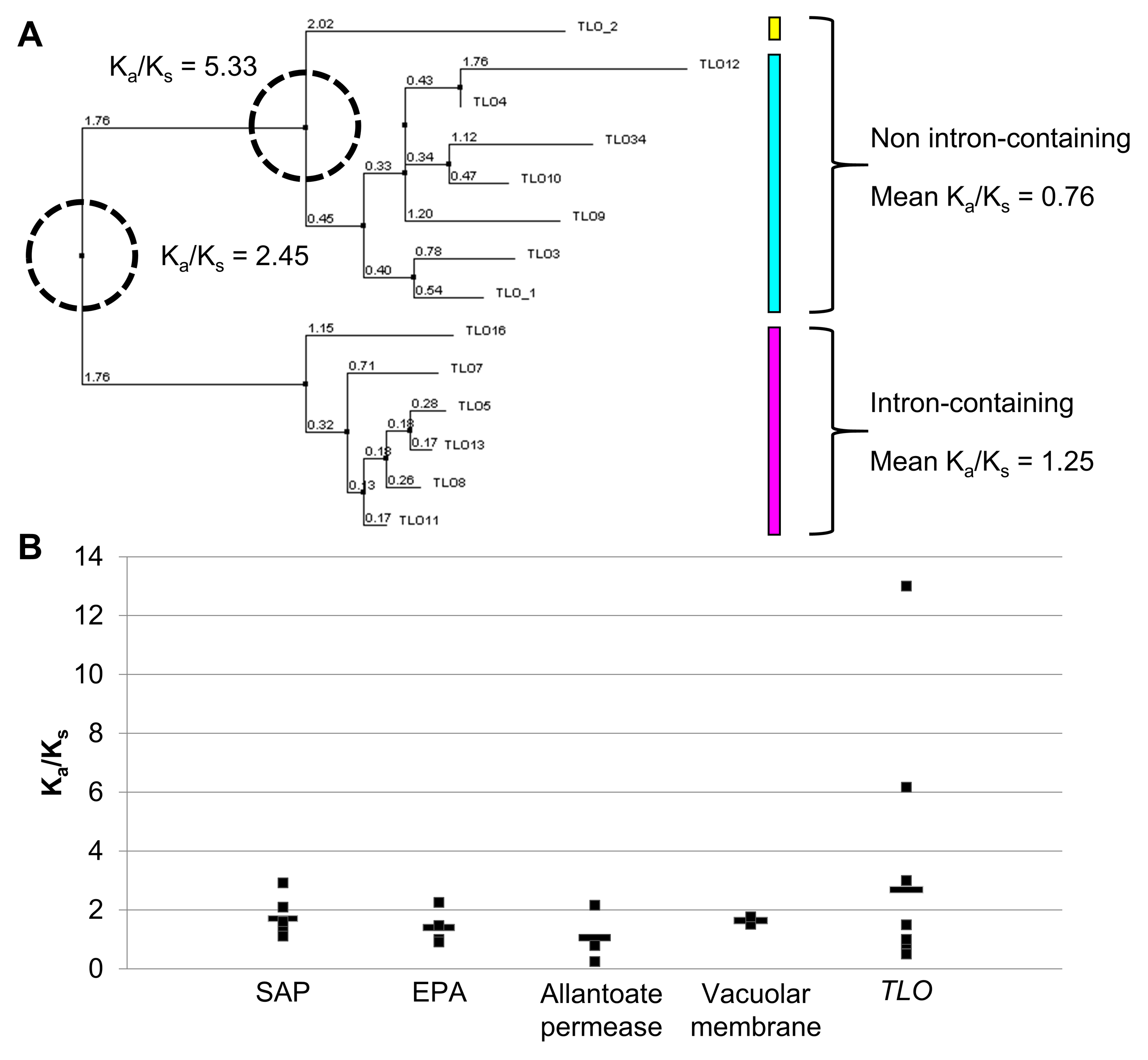

Supplement: S11 Fig — A. Selection coefficients (Ka/Ks) were calculated for all branch points within the TLO phylogeny. Major branch points separating the TLO clades (circled) possess exceptionally high Ka/Ks values whereas intra-clade branch points have more neutral selection coefficients. B. Selection coefficients were determined across expanded C. albicans gene families (SAP, allantoate permease, vacuolar membrane, TLO) and C. glabrata (EPA) for all nodes within their respective phylogenies. All gene families show evidence of positive selection with TLOs exhibiting the greatest effect of selection. (TIF) [file pgen.1007326.s011.tif]

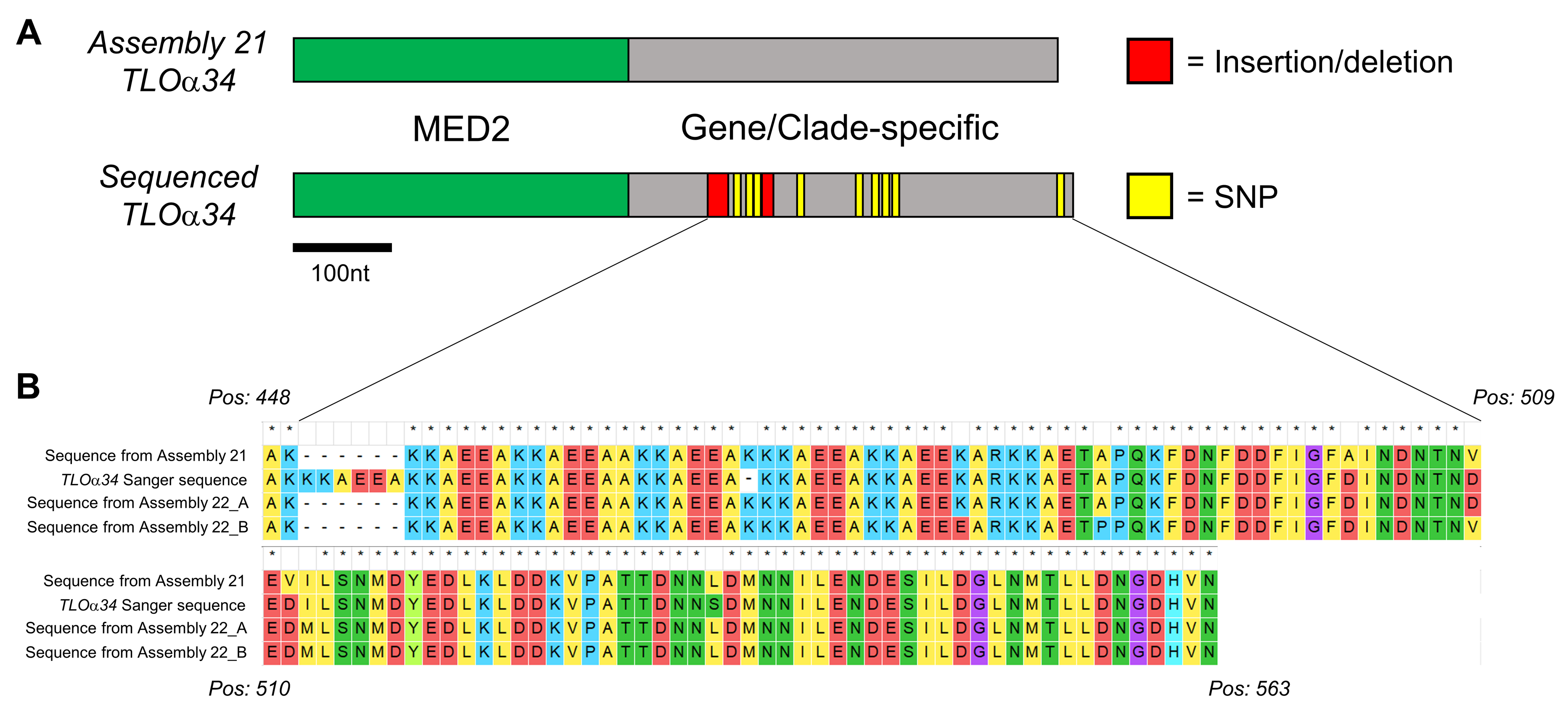

Supplement: S12 Fig — A. Polymorphic sites identified between the Sanger sequenced TLOα34 sequence used in this study and Assembly 21 (A21) are plotted across the gene. SNPs are highlighted in yellow and indels are highlighted in red. B. The resulting protein sequences from A21, both homologs in A22, and our Sanger sequenced TLO𝛂34 are aligned for comparison. Stars indicate identical positions and dashes indicate indels. (TIF) [file pgen.1007326.s012.tif]

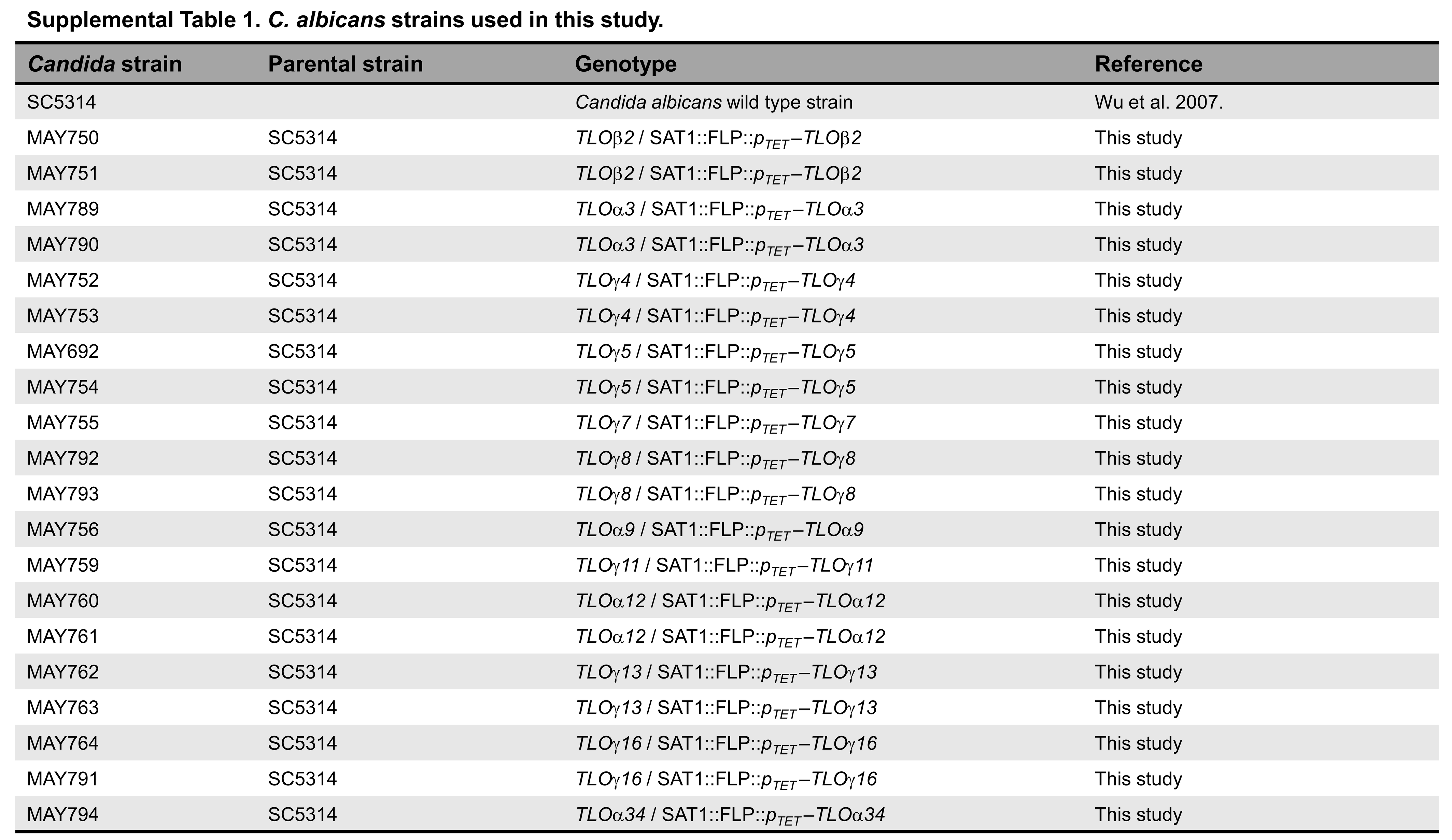

Supplement: S1 Table — (TIF) [file pgen.1007326.s013.tif]

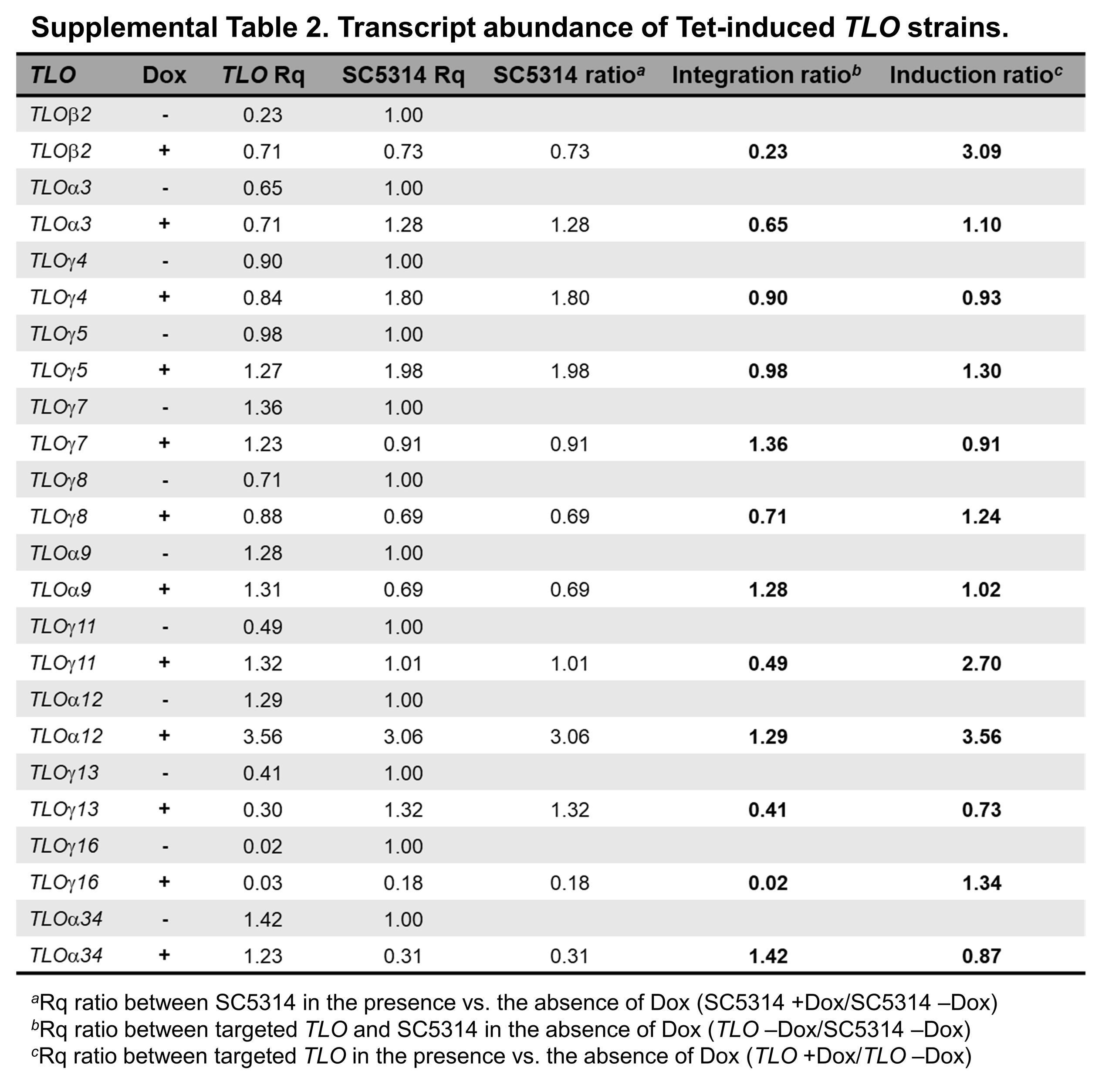

Supplement: S2 Table — (TIF) [file pgen.1007326.s014.tif]

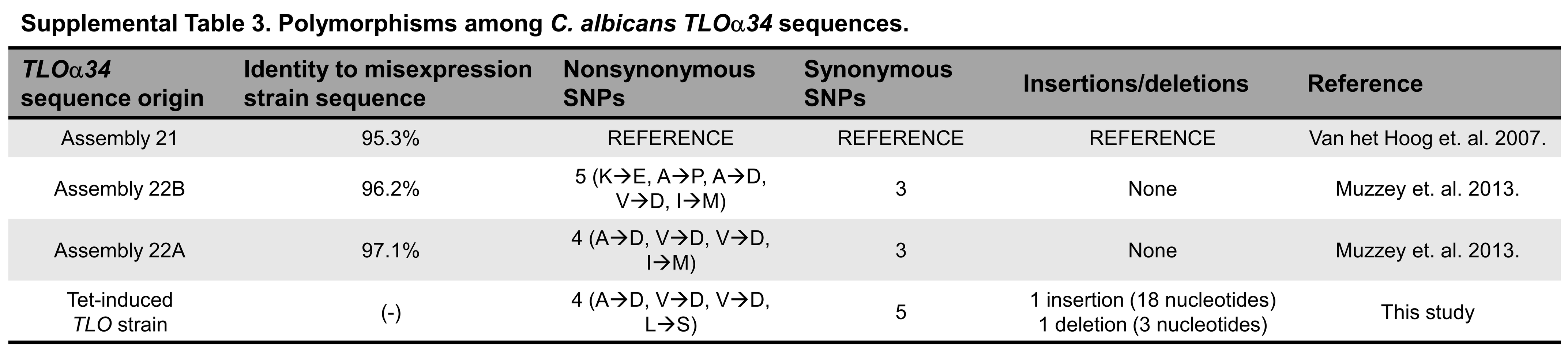

Supplement: S3 Table — (TIF) [file pgen.1007326.s015.tif]

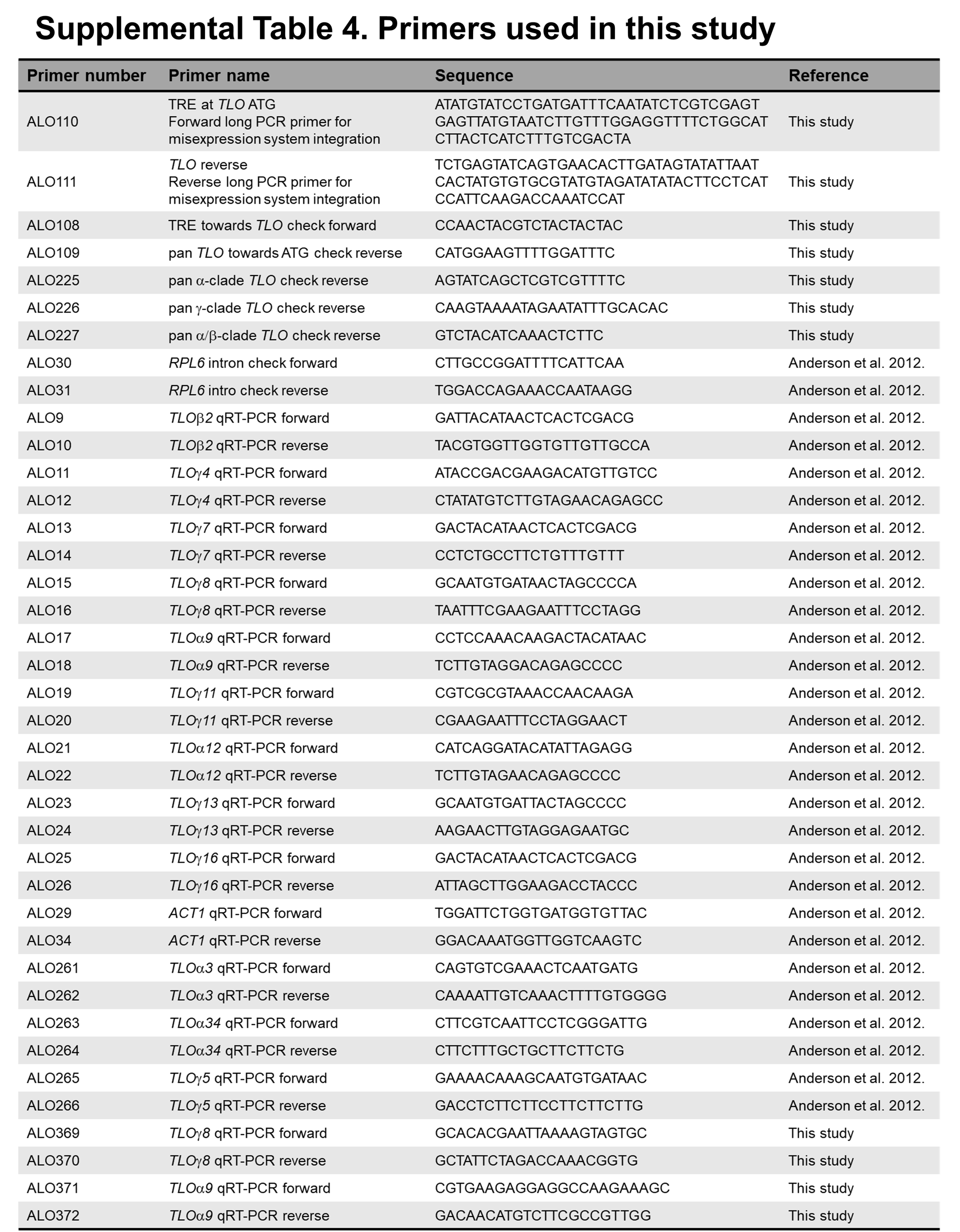

Supplement: S4 Table — (TIF) [file pgen.1007326.s016.tif]
